# Uncovering subgroup heterogeneity in dyslexia intervention outcomes using explainable machine learning

**DOI:** 10.1038/s41539-026-00429-3

**Published:** 2026-05-25

**Authors:** Amanda Swee-Ching Tan, Farhan Ali

**Affiliations:** Learning Sciences and Assessment, National Institute of Education, Nanyang Technological University, Singapore, Singapore

**Keywords:** Health care, Psychology, Psychology

## Abstract

Identifying heterogeneity within literacy intervention outcomes can inform more targeted strategies for dyslexia remediation. Based on prior work that used machine learning to predict literacy intervention responders and non-responders at baseline, this study uncovered 2 subgroups of responders and 4 subgroups of non-responders based on feature importance profiles of 132 students who were diagnosed with dyslexia. Their average age was 8.2 years at baseline (98.08 months). They received literacy intervention for an average of 33.62 months. Intervention subgroup membership predicted long-term literacy development of a subsample of 41 students 68.2 months post intervention. The baseline scores, top predictors and long-term literacy outcomes were profiled for each subgroup. *‘High Baseline Responders*’ and ‘*Low Baseline Responders*’ characterized the two responder subgroups. *‘Total Regression Non-Responders’*, ‘*Self-Taught Non-Responders’*, ‘*Phonics-Receptive Non-Responders’* and *‘Lowest Baseline Non-Responders’* characterized the four non-responder subgroups. Overall, our research found that clustering could identify subgroup profiles and demonstrates the predictive potential of longitudinal literacy development.

## Introduction

Individuals who are at-risk for or diagnosed with dyslexia face increased risk of adverse life outcomes that are associated with poor literacy skills^1^. Apart from financial costs, low literacy is also a social determinant of poor health outcomes^2^. Individuals with low literacy skills face elevated risk of dementia and disease, and an earlier onset of mortality^3,4^. The cost of dyslexia resulted in efforts to understand the underlying causes to mitigate the consequences. Multiple theories of dyslexia have emerged in response to the population heterogeneity. The theories include the phonological deficit^5^, double deficit^6^, magnocellular deficit^7^ and cerebellar deficit^8^. The phonological deficit theory focuses on the deficit in phonological processing of words, while the double deficit theory focuses on both deficits in phonological processing and rapid naming. The cerebellar deficit theory and magnocellular deficit theory focuses on the motor coordination and automatization deficits, and visual motion perception deficits, respectively. Among the different theories of dyslexia, the prevailing literacy intervention for poor readers and spellers is highly influenced by the phonological deficit theory^9,10^. Due to the heterogeneity of profiles among individuals with dyslexia, some poor readers and spellers fail to improve despite receiving phonic-based literacy intervention (e.g., Orton Gillingham). The prevalence of literacy non-responsiveness can reach as high as 50%^11^ with 92% of non-responsive kindergarteners demonstrating persistent non-responsiveness into first grade^12^.

Prior prediction of intervention non-responsiveness would potentially mitigate the above risks and encourage the provision of customized support for the non-responders. However, there are three constraints. First, the evaluation of intervention is typically restricted to classical predictors and outcomes (e.g., phonological awareness, rapid naming) that are dependent on the phonological deficit theory due to prior assumptions of symptom homogeneity and localized linguistic neural systems^13^. Second, the short-term nature of interventions, together with the measure of responsiveness based on near-transfer outcomes (i.e., pseudoword decoding, phonemic awareness), risk bias toward a phonological explanation without accurately reflecting the diversity of cognitive profile of responders and non-responders. For instance, difference in far transfer outcome orthographic measures between responders and non-responders after receiving the phonics intervention suggests the presence of intact orthographic processing of the former^14^. Third, the intervention evaluations that achieve adequate sample size and intervention duration are typically Dutch instead of the English language interventions^15,16^. Unlike English, Dutch has a transparent orthography and consistent mappings between symbol and sound. The findings based on the intervention of transparent orthographies may not be directly generalizable to opaque languages like English, which contains inconsistent letter-sound correspondence with many rule exceptions.

Contrary to assumptions of localized neural systems that are relevant to language functioning, emerging research has shown that a diverse array of neural subsystems is involved in the cognitive processes of literacy acquisition. These cognitive functions include phonological, morphological, orthographic, and semantic processing, all of which differentiated yet interconnected neural networks. In particular, orthographic word-form processing has been found to predict nearly every measure of literacy achievement^17^. The evaluation of intervention responsiveness should prioritize far-transfer outcomes to determine literacy responsiveness, ensure extended durations of English language intervention and construct a dataset that encompasses a wider array of predictors, extending beyond the conventional parameters rooted in phonological theory. To date, only two studies have built a model to predict responsiveness to literacy at baseline that incorporated these considerations. In the first study^18^, a five-stage hierarchical logistic (linear) regression was trained with 12 baseline and demographic predictors of adequate responders (*n* = 35) and persistently poor responders (*n* = 80) who underwent 24 months of phonics-based intervention^18^. While persistently poor responders underperformed across all measures, far transfer output measures (i.e., reading: *η2* = 0.08; spelling: *η2* = 0.06) were worse than near transfer output measures (e.g., pseudoword decoding: *η2* = 0.28). Phonological awareness and listening-reading comprehension discrepancy did not predict response to intervention. The F1 score of approximately 85% was computed only on training data. Conversely, a second study^14^ trained a semi-supervised learning model with 196 discrepancy predictors based on a broader set of cognitive processes (e.g., spatial reasoning) that are involved in the literacy acquisition of 849 poor readers and spellers. Specifically, the dataset included the baseline and post-intervention scores of 132 participants with dyslexia who received an average of 33.6 months of intervention. The rest of the dataset were participants who only had baseline scores and did not receive intervention but were included via semi-supervised machine learning, which can handle both labeled and unlabeled data. Literacy responsiveness was determined using far-transfer measures (i.e., reading, spelling) of the 132 participants. The predictive model was trained on both types of data to ensure generalizability across different settings and to reflect the reality of the education setting in schools. The best performing F1 score on the hold-out test set was 72% while the training data yielded a score of 91%.

With existing research predicting binary classes of literacy responsiveness, there is potential in different subgroup profiles within each class to facilitate personalized instruction. The different subgroups reflect the diverse cognitive and linguistic processes involved in the language acquisition of a heterogeneous population despite similar short-term literacy outcomes. Apart from post-intervention responsiveness and subgroup membership, prediction using baseline data should also chart literacy trajectory at follow-up (i.e., a period of time after intervention) after the completion of intervention. Longitudinal outcomes are essential to create strong subgroup profiles and determine the sustenance of post-intervention findings. In addition, strong predictors of future outcomes can be identified with confidence and delayed effects that only emerge months or years later can be uncovered in advance. Furthermore, individual variability can also be accounted for as the same participants are tracked over time. At present, the findings on literacy interventions evaluation research for dyslexia are unable to sustain over time. 71 literacy interventions that averaged 11.7 months of follow-up measurements saw decreased effect sizes with only a small effect maintained in the long term (short-term effect: d = 0.37; follow-up test: d = 0.22), with phonics intervention producing the most short-lived effectiveness^19^. The findings underscore the necessity of long-term data to validate the long-term effect of literacy responsiveness.

A combination of model explainability with clustering facilitates better understanding of complex, multivariate machine learning models. A model explainer can identify the predictors that contributed to the model decision regarding class membership. One such explainer framework is the Local Interpretable Model-Agnostic Explanations (LIME)^20^. LIME perturbs the input data locally around a single instance and fits a simple interpretable model (e.g., linear regression) in the surrounding area of predictive interest. To account for the tendency of explainer models to overstate class homogeneity due to such local approximations, explanation-aware clustering via unsupervised learning could be used to identify heterogeneous subgroups within each class to facilitate the simultaneous process of model transparency alongside the uncovering of heterogeneity within a dataset^21^. Importantly, the clustered subgroups have the potential to predict longitudinal outcomes, i.e., not just of intervention responsiveness but also long-term literacy trajectories years after intervention. Cognitive, achievement and/or behavioral data from psychological assessments can be fed into unsupervised machine learning algorithms (e.g. K-means, Self Organising Map) to allow objective clustering of data - irrespective of diagnosis - and establish cognitive commonality before interpreting subgroup characteristics and profiles. For instance, a study^22^ identified four subgroups of subjective well-being profiles among children with special educational needs. The ‘socializer’ and ‘analyzer’ subgroups were characterized by interpersonal functioning and academic skills predictors, respectively. The remaining two subgroups consisted of demographic and other diverse predictors. Another study^23^ clustered 223 Mandarin-speaking children diagnosed with dyslexia into four clusters based on measures of phoneme deletion, rapid naming and morphological awareness: (C1) Global deficit; (C2) Phonological deficit; (C3) Rapid naming deficit; and (C4) Mild morphological deficit.

Data-driven clustered subgroups are also can also be examined for predictive validity via longitudinal data where the outcomes of each subgroup can be examined for unique predictors or differentiating trajectory patterns. A study^24^ found three clusters from a population of 828 children with no diagnosis and those who were diagnosed with ADHD. 3 clusters were identified based on temperament - (1) Surgent, (2) Irritable, and (3) Mild. Through longitudinal data, cluster membership of 2 [Irritable] and 3 [Mild] were predicted to have higher risk for new onset of anxiety diagnoses. Another study^25^ attempted to explore the predictive validity of 3 clusters within a population of 1098 children diagnosed with autism 3 clusters: (C1) High functioning strata, (C2) Medium functioning strata, and (C3) Low functioning strata. Clustering performed with a dataset of children’s later years reproduced the clusters of the early years dataset with an external validity of 99% generalization accuracy. In addition, C1 and C3 are likely to remain in the same functioning stratum in the later years, while C2 has more varied outcome membership of high, relatively low and outliers. While promising work in neighboring areas of neurodevelopmental disorders have uncovered subgroups with a diversity of profiles using long-term outcomes, this approach has not been used to uncover profiles in children with developmental dyslexia who have undergone intervention.

Existing research has demonstrated the prediction of literacy intervention responsiveness using the baseline data of individuals with or at-risk of dyslexia^14,18^. However, research on clustered subgroups and their associated longitudinal outcomes based on predicted classes of responsiveness remains unexplored. To demonstrate the predictive capability of machine learning in uncovering meaningful heterogeneity in dyslexia intervention outcomes, this study’s research objectives are:

1. To cluster individuals based on Local Interpretable Model-Agnostic Explanation (LIME) profiles to identify distinct subgroups within responder and non-responder groups
2. To examine the longitudinal achievement outcomes of each subgroup to assess long-term literacy progression

## Results

### Research objective 1

**Fig. 1:**
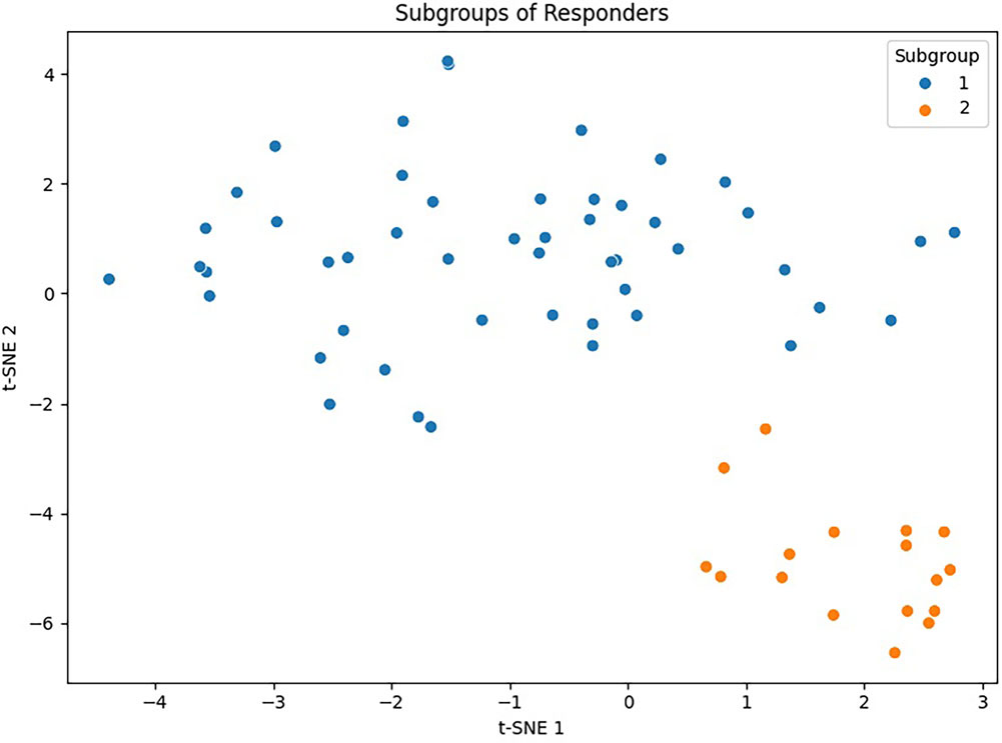
K-means clustering of responder subgroups (*k* = 2). LIME coefficients of 196 baseline and pairwise difference predictors for responders were clustered via K-Means and featured in a dimensionally reduced visual of two axes via t-SNE.

**Fig. 2:**
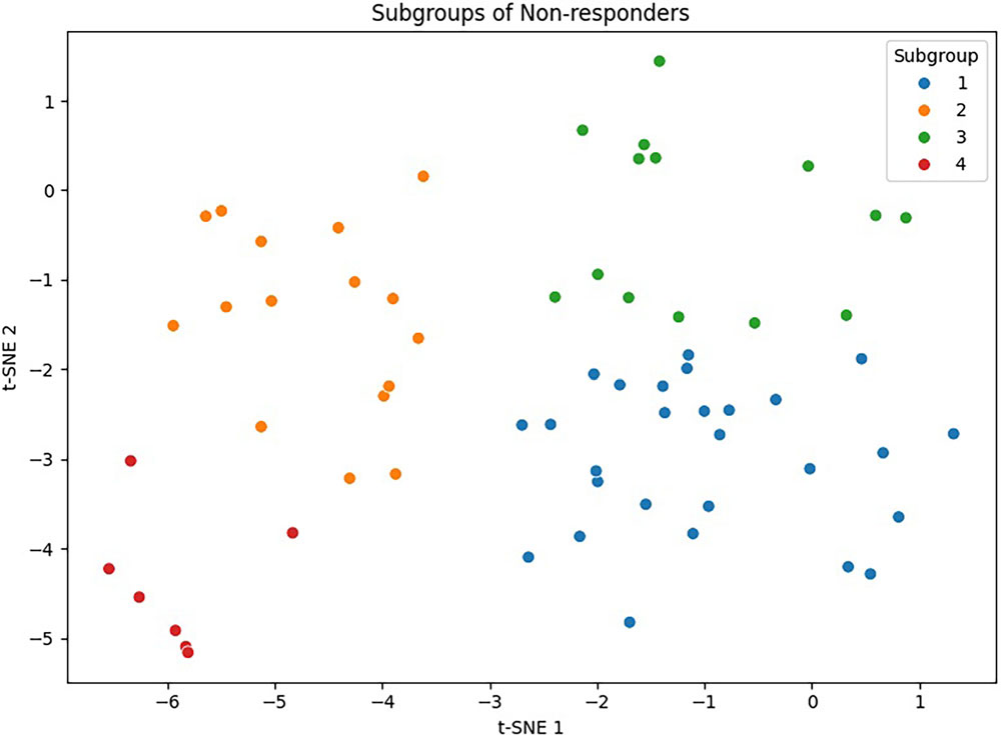
K-means clustering of responder subgroups (*k* = 4). LIME coefficients of 196 baseline and pairwise difference predictors for non-responders were clustered via K-Means and featured in a dimensionally reduced visual of two axes via t-SNE.

To uncover the heterogeneous profiles in the responder and non-responder groups that were identified in a previous study^14^, we used the Davies–Bouldin Index (DBI) to evaluate the optimal number of clusters formed via K-Means. Two subgroups were identified under the responder group (DBI = 0.81), and four subgroups were identified under the non-responder group (DBI = 0.48). The final solutions reported (DBI = 0.81 for responders; DBI = 0.48 for non-responders) correspond to the lowest DBI. This means that the within-cluster variation is the smallest compared to between-cluster separation, i.e., the clusters were most distinct overall. The empirical criterion was further supported by visual assessment showing separate clusters, providing justification for the chosen clustering solutions. Figures 1 and 2 show the clustering of responder and non-responder subgroups. Responder Subgroup 1 is comprised of 48 participants, while Responder Subgroup 2 is comprised of 17 participants. Non-Responder Subgroup 1 is comprised of 27 participants. Non-Responder Subgroup 2 is comprised of 18 participants. Non-Responder Subgroup 3 is comprised of 15 participants. Non-Responder Subgroup 4 is comprised of 7 participants. Membership and the associated predictor values of each cluster were collated into smaller datasets. The values for each predictor were averaged per cluster. The predictors were ranked according to descending order of predictive contribution in each cluster. The mean discrepancy score for each predictor was compared against the global average of to be labeled either ‘high’ or ‘low’. Table 1 shows the shared predictors for the responder subgroups. Table 2 shows the shared predictors for the non-responder subgroups. Supplementary Table 2 shows the LIME feature interpretation table. Supplementary Table 3 shows the subgroup comparisons of average baseline measures per responder subgroup. Supplementary Table 4 shows the subgroup comparisons of average baseline measures per non-responder subgroup. Supplementary Table 5 shows the Mann–Whitney Test comparing the longitudinal outcomes of subgroups (p-values adjusted for multiple comparisons using FDR). Supplementary Table 6 shows the effect sizes corresponding to Mann–Whitney Test for longitudinal outcomes of subgroups. Supplementary Table 7 shows the complete list of metrics in the dataset.

**Table 1:** Top 5 shared predictors for the responder subgroups

Subgroup 1
Gestation (weeks) (−)
Memory: Recall of Picture (−)
Duration of Early Intervention (months) (+)
Memory: Recall of Object (Immediate-average) (−)
Verbal Comprehension: Similarities (+)
Subgroup 2
Gestation (weeks) (−)
Memory: Recall of Picture (−)
Duration of Early Intervention (months) (−)
Verbal Comprehension: Similarities (+)
Digit Span: Average (−)

Note: (+) = score contributed positively to the predicted probability of the respective responder subgroup. (−) = score contributed negatively to the predicted probability of the respective responder subgroup.

**Table 2:** Top 5 Shared Predictors for the Non-responder Subgroups

Subgroup 1
Gestation (weeks) (+)
Memory: Recall of Picture (+)
Duration of Early Intervention (months) (−)
Memory: Recall of Object (Immediate Average) - Verbal Comprehension: Similarities (+)
Memory: Recall of Object (Immediate Verbal) - Verbal Comprehension: Similarities (+)
Subgroup 2
Gestation (weeks) (+)
Memory: Recall of Object (Immediate Spatial) (+)
Memory: Recall of Object (Immediate Average) (+)
Memory: Recall of Object (Delayed Verbal) - Memory: Recall of Object (Delayed Average) (+)
Verbal Comprehension: Similarities (−)
Subgroup 3
Gestation (weeks) (+)
Memory: Recall of Picture (+)
Memory: Recall of Object (Immediate Average) - Verbal Comprehension: Similarities (+)
Duration of Tier 1 Early intervention (months) (+)
Memory: Recall of Object (Immediate Verbal) - Verbal Comprehension: Similarities (+)
Subgroup 4
Gestation (weeks) (−)
Duration of Early Intervention (months) (+)
Nonverbal: Sequential and Quantitative Reasoning - Memory: Recall of Object (IS) (+)
Digit Span: Average - Memory: Recall of Object (IS) (+)
Memory: Recall of Picture (+)

Note: (+) = score contributed positively to the predicted probability of the respective non-responder group. (−) = score contributed negatively to the predicted probability of the respective non-responder group.

Despite variation in the sign and impact of its contribution, the gestation and recall of objects was consistently among the top-ranked contributors identified by LIME across all subgroups. Reduced (<39.18 weeks) and increased gestational duration (>39.18 weeks) were positive contributors to all responder and non-responder groups respectively. Low memory scores for immediate recall of objects (Immediate Average: <97.66 Standard Score (SC)); Immediate Spatial: <95.35 SC) and high memory scores for delayed verbal recall of objects (Delayed Verbal – Delayed Average: >0.42 SC) were positive contributors to the responder group.

### Top predictors per subgroup

In Responder Subgroup 1 (RSG1), long duration of gestation (39.84 weeks), high recall of picture scores (ROP: 97.91 SC) and low recall of object scores (Immediate Average (IA): 96.76 SC) were negative contributors to the predicted probability of the RSG1. Short duration of early intervention (4.37 months) and high verbal comprehension scores (Similarities = 98.74 SC) were a positive contributor to the predicted probability to the RSG1.

In Responder Subgroup 2 (RSG2), short duration of gestation (35.24 weeks) and low recall of object scores (IS = 84.76 SC, IA = 81.83 SC) were positive contributors to the predicted probability of the RSG2. High scores of recall of object disparity measures (DV - DA = 1.28 SC) and low verbal comprehension scores (Similarities = 89.57 SC) were a positive contributor to the predicted probability of RSG2.

In Non-responder Subgroup 1 (NRSG1), long duration of gestation (40 weeks), high memory recall of picture = 100.88 SC), high scores of memory-verbal comprehension similarities disparity (IA - Similarities = 20.62 SC, IV - Similarities = 8.54 SC) were positive contributors to the predicted probability of the non-responder group. The short duration of early intervention negatively contributed to the predicted probability of the non-responder group.

In the Non-responder Subgroup 2 (NRSG2), long duration of gestation (40.06 weeks), high scores of memory recall of object (IS = 110.27 SC, IA = 97.86 SC) and low memory disparity scores (DV – DA) (−7.80) positively contributed to the predicted probability of the non-responder group. The high verbal comprehension score (Similarities = 89.72 SC) contributed negatively to the predicted probability of the NRSG2.

In the Non-responder Subgroup 3 (NRSG3), long duration of gestation (39.94 weeks), long duration of early intervention (20.25 months), high memory-verbal comprehension disparity scores (IA - Similarities = 13.78 SC, IV - Similarities = 15.70 SC) contributed positively to the predicted probability of the NRSG3.

In the Non-responder Subgroup 4 (NRSG4), short duration of gestation (37.14 weeks) contributed negatively to the predicted probability of NRSG4. Long duration of early intervention (37.14 months), low recall of picture scores (ROP = 90.14 SC), low nonverbal-recall of object disparity scores (SQR –IS = −14.43 SC), low digit span-recall of object disparity scores (Average - IS = −19.93 SC) contributed positively to the predicted possibility of the NRSG4.

### Subgroup comparisons of baseline measures

Beyond the top predictors, comparisons between the baseline measures of responder subgroups reveal that Responder Subgroup 1 (RSG1) has overall higher baseline measures as compared to Responder Subgroup 2 (RSG2). This is evident in the following measures: (1) Literacy (RSG1 Reading = 91.26 SC, RSG2 Reading = 82.61 SC|RSG1 Spelling = 92.39 SC, RSG2 Spelling = 81.59 SC), (2) Speed of Processing (RSG1 SP = 106.80 SC, RSG2 SP = 93.24 SC), (3) Verbal Comprehension Word Definitions (RSG1 WD = 96.72 SC, RSG2 WD = 91.73 SC), (4) Digit Span (RSG1 Forward = 111.52 SC, RSG2 Forward = 83.00 SC|RSG1 Backwards = 94.00 SC, RSG2 Backwards = 81.83 SC|RSG1 Average = 102.77 SC, RSG2 Average = 82.41 SC), (5) Spatial (RSG1 Pattern of Construction (POC) = 102.95 SC, RSG2 POC = 88.88 SC|RSG1 Recall of Design (ROD) = 103.04 SC|RSG2 ROD = 88.00 SC), and (6) Non Verbal (RSG1 Matrix = 103.62 SC, RSG2 Matrix = 80.37 SC|RSG1 Sequential and Quantitative Reasoning (SQR): 105.41 SC, SQR = 84.68 SC).

Comparisons between the baseline measures of non-responder subgroups reveal various patterns. Firstly, Non-responder Subgroup 1(NRSG1) and Non-responder Subgroup 2 (NRSG2) received much shorter durations of early intervention as compared to Non-responder Subgroup 3 and Non-responder Subgroup 4 (NRSG1 = 3.70 months, NRSG2 = 7 months, NRSG3 = 20.25 months, NRSG4 = 37.14 months). Secondly, the literacy performance across all subgroups is similar (NRSG1 Reading = 80.06 SC, NRSG2 Reading = 81.44 SC, NRSG3 Reading = 80.73 SC, NRSG4 Reading = 83.36 SC|NRSG1 Spelling = 84.02 SC, NRSG2 Spelling = 85.11 SC, NRSG3 Spelling = 82.29 SC, NRSG4 Spelling = 80.71 SC). Thirdly, NRSG2 and NRSG4 demonstrate higher word definition scores as compared to NRSG1 and NRSG3 (NRSG1 WD = 74.58 SC, NRSG2 WD = 100.89 SC, NRSG3 WD = 79.32 SC, NRSG4 WD = 91.57 SC).

In addition, NRSG1 and NRSG2 demonstrate higher speed of information processing scores (NRSG1 SIP = 107.41 SC, NRSG2 SIP = 100.72 SC) and immediate recall of object (NRSG1 IV = 108.51 SC, NRSG2 IV = 97.86 SC|NRSG1 IS = 96.00 SC, NRSG2 IS = 110.27 SC) as compared to NRSG3 and NRSG4 (NRSG3 SIP = 94.76 SC, NRSG4 SIP = 94.57 SC|NRSG3 IV = 96.06 SC, NRSG4 IV = 99.29 SC|NRSG3 IS = 94.98 SC, NRSG4 IS = 98.71 SC). Conversely, NRSG3 has comparatively lower delayed recall of object scores (NRSG3 DV = 81.83 SC|NRSG3 DS = 93.66 SC) as compared to the other subgroups (NRSG1DV = 107.86 SC, NRSG2 DV = 102.52 SC, NRSG4 DV = 104.43 SC|NRSG1 DS = 96.88 SC, NRSG2 DS = 111.83 SC, NRSG4 DS = 102.29 SC).

Lastly, NRSG4 has comparatively lower non-verbal (NRSG4 Matrix = 82.00 SC, SQR = 84.29 SC), spatial (NRSG4 POC = 95.29 SC, ROD = 93.14 SC) and digit span scores (NRSG4 Forward = 79.86 SC, Backwards = 77.71 SC,) as compared to the other subgroups (NRSG1 Matrix = 100.39 SC, NRSG2 Matrix = 100.39 SC, NRSG3 Matrix = 97.31 SC|NRSG1 SQR = 97.42 SC, NRSG2 SQR = 97.04 SC, NRSG3 SQR = 94.91 SC|NRSG1 POC = 104.05 SC, NRSG2 POC = 105.11 SC, NRSG3 POC = 101.66 SC|NRSG1 ROD = 103.63 SC, NRSG2 ROD = 106.74 SC, NRSG3 ROD = 101.19 SC|NRSG1 Forward = 88.82 SC, NRSG2 Forward = 97.74, NRSG3 Forward = 96 SC|NRSG1 Backwards = 91.84 SC, NRSG2 Backwards = 93.72 SC, NRSG3 = 87.78).

### Research objective 2

To examine the longitudinal achievement outcomes of each subgroup, we recruited 41 participants across all subgroups. Due to the retrospective nature of the previous study, recruitment was uneven across the subgroups. Responder Subgroup 2 and Non-Responder subgroup 4 were omitted in the analysis due to small sample sizes (*n* = 1). The 39 participants from the remaining subgroups underwent testing at T2. The subgroups showed distinct profiles based on longitudinal outcomes. Table 3 shows the average literacy scores at T2 for responder and non-responder subgroups along with sample sizes. Table 4 shows the directionality of the longitudinal achievement task performance across all subgroups. Supplementary Table 5 shows the Mann–Whitney Test scores comparing the longitudinal outcomes of subgroups. Supplementary Figs. 1–8 shows the longitudinal word reading, word spelling, pseudoword decoding and reading comprehension scores of responder subgroup 1 and non-responder subgroups 1, 2 and 3.

**Table 3:** Average literacy scores at T2 for responder and non-responder subgroups

Tests	Responder subgroup 1 (*n* = 19)	Non-responder Subgroup 1 (*n* = 13)	Non-responder Subgroup 2 (*n* = 3)	Non-responder Subgroup 3 (*n* = 4)
∆ T1–T0 standard scores				
Word reading	9.06 (1.67)	−8.08 (2.03)	−9 (7.02)	−7.5 (1.76)
Word spelling	10 (2.05)	−7.31 (1.96)	−3.33 (7.22)	−7.75 (2.32)
Pseudoword decoding	4.89 (2.42)	−10.62 (2.99)	−24.67 (2.73)	1.5 (6.24)
Reading comprehension	8.94 (2.26)	1.31 (4.24)	−6.67 (10.81)	3.5 (3.10)
∆ T2–T1 standard scores				
Word reading	0.39 (1.99)	−1.77 (2.12)	3 (3.51)	9 (0.41)
Word spelling	2.06 (1.79)	−0.54 (1.55)	1 (2.31)	7.5 (1.32)
Pseudoword decoding	0.72 (2.39)	−6.92 (3.81)	16.67 (11.86)	−5 (1.87)
Reading comprehension	−11.56 (3.70)	−13.46 (4.03)	−2.67 (7.06)	−9.5 (5.89)

**Table 4:** Directionality of achievement task performance across all subgroups

Tests	Responder subgroup 1	Non-responder subgroup 1	Non-responder subgroup 2	Non-responder subgroup 3
Reading	T0–T1: Improve^a^	T0–T1: Regress^b^	T0–T1: Regress^b^	T0–T1: Regress^b^
	T1–T2: Maintain^a^	T1–T2: Regress^b^	T1–T2: Regress^b^	T1–T2: Improve^a^
Spelling	T0–T1: Improve^a^	T0–T1: Regress^b^	T0–T1: Regress^b^	T0–T1: Regress^b^
	T1–T2: Improve^a^	T1–T2: Regress^b^	T1–T2: Improve^b^	T1–T2: Improve^a^
Pseudoword decoding	T0–T1: Improve^a^	T0–T1: Regress^b^	T0–T1: Steep Regression^b^	T0–T1: Improve^a^
	T1–T2: Maintain^a^	T1–T2: Regress^b^	T1–T2: Steep Improvement^b^	T1–T2: Regress^b^
Reading comprehension	T0–T1: Improve^a^	T0–T1: Improve^a^	T0–T1: Regress^b^	T0–T1: Improve^a^
	T1–T2: Regress^b^	T1–T2: Regress^b^	T1–T2: Regress^b^	T1–T2: Regress^b^

Improve = 1–15 | Regress = <0 | Maintained = 0≤/<1 | Steep = >+15/<−15.

^a^Above baseline.

^b^Below baseline.

Responder Subgroup 1 exhibited a positive response to intervention across all measures (WR = 9.06; WS = 10; PD = 4.89; RC = 8.94) and maintained their performance over the long-term following the intervention across all measures (WR = 0.39; WS = 2.06; PD = 0.72) except reading comprehension, where a regression was observed (RC = −11.56).

Non-Responder Subgroup 1 exhibited a negative response to the intervention across all measures (WR = −8.08; WS = −7.31; PD = −10.62) except reading comprehension, where a marginal improvement was observed (RC = 1.31). The subgroup exhibited a further regression over the long-term following the intervention (WR = −1.77; WS = −0.54; PD = −6.92; RC = −13.46).

Non-Responder Subgroup 2 exhibited a negative response to the intervention across all measures (WR = −9; WS = −3.33; PD = −24.67; RC = −6.67) but exhibited an improvement across all measures (WR = 3; WS = 1; PD = 16.67), except reading comprehension (RC = −2.67), over the long-term following the intervention.

Non-Responder Subgroup 3 exhibited a negative response to the intervention across reading and spelling measures (WR = −7.5; WS = −7.75) while marginal improvements were demonstrated in pseudoword decoding and reading comprehension (PD = 1.5; RC = 3.5). The subgroup exhibited an inverse performance across all measures over the long-term following the intervention. Reading and spelling measures (WR = 9; WS = 7.5) showed an improvement, while pseudoword decoding and reading comprehension (PD = −5; RC = −9.5) regressed over the long term.

## Discussion

This study clustered individuals based on LIME profiles to identify distinct subgroups within literacy intervention responder and non-responder groups. Top predictors for membership of each subgroup were identified. In addition, the subgroups, based solely on the collection of predictor importance (not actual predictor values directly), were also able to demonstrate subsequent predictive potential of the longitudinal outcomes for Responder Subgroup 1, Non-responder Subgroup 1, 2 and 3. Long-term literacy progression was charted and the directionality of performance across all achievement measures differentiated the subgroups. The top predictors, baseline characteristics and long-term progression outcomes formed the profiles of each subgroup. Membership of all responder subgroups was predicted by short gestation period, high verbal comprehension (similarities) scores and generally low immediate recall of object and picture. Membership of all non-responder subgroups was predicted by low baseline verbal comprehension (similarities) and scores. All non-responder subgroups except Non-Responder Subgroup 4 are positively predicted by long gestation and high immediate recall of object scores.

It is known that gestational age at delivery has a correlation with the risk of developing complications including special educational needs (SEN). For instance, pre-mature babies are at much higher risk of developing SEN^26^. However, there seemed to be assumed linearity in the relationship between gestational age and the risk of SEN or its associated complexity, in which lowered risk of SEN surfaces with longer the gestational age. However, this study found that longer gestation (>39.18 weeks) predicted literacy non-responsiveness while comparatively shorter gestation of 35–39 weeks predicted literacy responsiveness. The findings relating to post-term gestation (i.e., >40 weeks) aligns with the consensus that the risk of SEN increases at post term, ranging from falling intelligence scores^27^ to an increased risk of developing intellectual disability^28,29^. The point of contention arises in the comparison between early-term births (i.e., 37–39 weeks) and full-term births (i.e., 40 weeks). As compared to their full-term counterparts, early-term births have been found to experience elevated risk of cognitive impairments^30,31^, improve cognitive performance with every succeeding week of gestation, or exhibit similar cognitive scores^32^. In comparing individual gestational weeks, a study^33^ found that while the prevalence of elevated risk of SEN is elevated for early-term births, the risk declined with every successive week but was nevertheless significantly elevated among children born at 39 weeks. The findings for early-term births in this study seem to accommodate these polarizing views by suggesting that while the children were diagnosed with SEN, the comparatively elevated cognitive abilities among early-term births - as compared to the non-responders that were predicted by longer gestation - may have resulted in increased responsiveness towards literacy intervention.

Responders have been found to possess stronger verbal reasoning abilities as compared to their non-responding counterparts. Students with dyslexia who have superior verbal reasoning outperform their counterparts with average or below average reasoning across all achievement and cognitive measures^34^. While they do not differ reliably from those with average reasoning in any measures across the literacy measures, these students seem to capitalize on their superior verbal reasoning abilities to aid in the morphological and syntactic aspects of literacy. However, these students continue to perform below the population mean in literacy despite not necessarily being the worst performing readers and spellers in their class. Indeed, Responder Subgroup 1 - the best performing subgroup in this study – still exhibits scores that are below the population mean.

The prevalence of strong visual memory among poor spellers or non-responders has been well documented in the literature, in which good visual memory recall of objects is not indicative of subsequent orthographic processing in word recognition as less efficient orthographic processing skills - not inferior visual memory - are characteristic of poor spellers^35,36^. In addition, good visual memory recall of objects has been found to be predictive of intervention non-responsiveness when it is presented in the presence of poor auditory working memory^14^. More importantly, the contrast between both responders and non-responders underscores the point that good visual memory recall of objects is not indicative of subsequent orthographic processing in word recognition. The clustered subgroups in this study were able to delve deeper into the different profiles of predictors. Non-responder subgroups 1–4 exhibit good memory for recall of objects, with the worst performing subgroup, NRSG1, demonstrating the strongest visual memory scores. Notably, NRSG 4 was the only subgroup with poor baseline auditory working memory scores that was predicted by good memory for object recall. These profiles suggest that poor auditory working memory may not be as significant a predictor of non-responsiveness as compared to strong visual memory. This is likely due to the limitations of visual memory in translating the representation of objects into symbolic recall and recognition via orthographic processing.

Within the responder class, Responder Subgroup 1 possesses statistically higher baseline scores as compared to subgroup 2 across all measures. Responder Subgroup 1 is uniquely differentiated by its prediction of high verbal comprehension (similarities) score. Conversely, Responder Subgroup 2 is uniquely predicted by low immediate recall of object scores and high delayed verbal recall of object scores offset by weak delayed spatial recall. Responder Subgroup 1 is characterized by an immediate recall of object score of 96.76 SC. All longitudinal achievement measures in Responder Subgroup 1 saw an improvement from T0 to T1. Reading and pseudoword decoding maintained at T2, while spelling improved and reading comprehension regressed.

Among the non-responder subgroups, Non-Responder Subgroups 1, 2 and 3 are similarly predicted by long duration of gestation and high immediate recall of object scores. The longitudinal reading outcomes from T0 to T1 similarly regressed across the subgroups. Despite such similarities among the subgroups, heterogeneity among subgroups is evident with unique predictors and the disparate directions of longitudinal outcomes among the other achievement tasks at follow-up. Unlike Subgroups 1 and 2, Non-Responder Subgroup 3 - and Subgroup 4 - are uniquely predicted by long duration of early intervention. In addition, Subgroup 1 is predicted by high recall of picture scores while subgroup 3 - and 4 - is predicted by relatively lower recall of picture scores. Non-Responder Subgroup 1 experienced regression across all measures and time points except reading comprehension at T1. Non-Responder Subgroup 2 experienced a regression across all time points for reading comprehension. Spelling regressed at T1 before improving at T2. Pseudoword Decoding saw a steep regression at T1 before experiencing a steep improvement at T2. Non-Responder Subgroup 3 experienced a regression in spelling at T1 before improving at T2. An inverse trend was evident among pseudoword decoding and reading comprehension, in which improvements and regressions were evident at T1 and T2, respectively.

The Responder Subgroup 1 that we have termed ‘*High Baseline Responders*’ represents students with high baseline cognitive profiles who exhibit strong verbal reasoning skills and compromised visual spatial abilities. The high baseline profile and strong verbal reasoning skills seemed to necessitate only minimal amount of early intervention. In addition, these students demonstrated receptivity to phonic intervention and demonstrated both near and far transfer improvements both at post and follow-up intervention. Improvements in pseudoword decoding also translated into corresponding improvements in reading and spelling. Conversely, Responder Subgroup 2, also known as the *‘Low Baseline Responders’*, necessitated longer periods of early intervention due to lower baseline cognitive profiles, suggesting earlier manifestations of literacy struggles at the preschool level.

The Non-Responder Subgroup 1 that we have termed *‘Total Regression Non-Responders’* represents the worst performing profile of students that experienced total regression across almost every achievement measure at T1 and T2. They had high baseline memory scores, especially in the recall of picture. These students did not receive much early intervention due to lack of early detection or the manifestation of dyslexia symptoms only encountering heightened academic demands in primary school. The slight improvement in reading comprehension at T1 seemed to suggest a compensatory strategy of attempting to understand - or guess - the meaning of text via context clues without necessarily being able to recognize the words. At T2, this compensatory ability was not sustained. This suggests that the inability to recognize enough words was critical enough to impede comprehension when the academic demands increased at the tertiary level, alongside the possible lack of motivation to read upon graduation. The top predictor of high recall of picture scores also suggests a strong inclination towards pictorial representations at the expense of cognitive functions that aid in the processing of symbolic word representations.

The Non-Responder Subgroup 2 that we have termed *‘Self-Taught Non-Responders’* represents the profile of students who exhibited confusion towards concept of phonics during the phase of intervention. This is evident by the steep decline in the near-transfer measures, pseudoword decoding, and regression in the far-transfer measures at T1. It is interesting to note that pseudoword decoding rebounded sharply while reading and spelling improved. These improvements occurred despite a lack of exposure to phonics intervention after T1. This development suggests that the academic demands years post-intervention necessitate these students to develop their preferred approach to literacy acquisition. Students with basic literacy demonstrate an increased reliance towards visual memory and strategies in attempt to remember whole words as a compensatory mechanism^37^. As such, improved literacy and pseudoword decoding scores at T2 suggest that their expanded word bank via allowed these students to ‘reverse engineer’ whole words and engage in independent learning of phonology. In other words, these students might have adopted a top-down approach to literacy acquisition. This understanding might have been supported by their high baseline scores for verbal comprehension (word definitions). However, it did not translate into improvements for reading comprehension, though the decline was lesser at T2 than it was at T1.

The Non-Responder Subgroup 3 that we have termed *‘Phonics-Receptive Non-Responders’* represents the profile of students who demonstrated only near-transfer improvements in pseudoword decoding during the phase of intervention. Conversely, far-transfer measures like reading and spelling regressed due to a lack of translation of gains from near to far transfer. Regardless, the students demonstrated increased comprehension via guided instruction despite regression in single-word recognition. This subgroup is also predicted by long duration of early intervention and low recall of picture scores. An inverse trend was observed at T2 where pseudoword decoding and reading comprehension regressed while reading and spelling improved. Taken together, the profile suggests the need for extensive early intervention due to low baseline scores. While the students demonstrated receptivity to phonological and morphological concepts, they were unable to generalize these concepts to whole word recognition and spelling. Like Non-responder Subgroup 2, the improvements in reading and spelling - at the expense of pseudoword decoding - occurred despite a lack of exposure to phonics after T1 probably due to the academic demands at the tertiary level necessitated the development of their preferred approach to literacy acquisition.

The Non-Responder Subgroup 4 that we have termed *‘Lowest Baseline Non-Responders’* is characterized by the profile with the lowest baseline scores that necessitates the longest duration of early intervention. Diverging from the profiles of the other non-responder subgroups, membership of this subgroup is predicted by long duration of early intervention, low immediate recall of object scores, high sequential and quantitative reasoning scores, and digit span scores. Membership predictors suggest initial inclinations towards numeracy and arithmetic instead of literacy.

The limitation of this study is the limited sample size involved in the analysis of longitudinal outcomes. Due to the retrospective nature of recruitment, the recruitment was limited by the absolute sample size and the random nature of subgroup membership. Non-Responder Subgroup 2 and 3 would have benefitted from a larger sample size. In addition, Responder Subgroup 2 and Non-responder Subgroup 4 were omitted from the analysis of longitudinal data due to low recruitment numbers. While it can be argued that low recruitment numbers are expected due to the small sizes of these subgroups in their respective responder and non-responder groups, the omission of these subgroups denies a more comprehensive view of the longitudinal trajectories of literacy outcomes of the clustered profiles across all subgroups. Future research should increase the sample size of each subgroup for more conclusive findings and confirm robust predictive utility. This is especially so when randomized recruitment could result in some subgroups having smaller sample sizes by chance, thereby underscoring the necessary to over-recruit to address this potential situation. Nonetheless, the recruitment of a sample that received extended intervention (i.e., 33 months) and prolonged delay of the follow-up assessment (i.e., average range of 63–71 months) remains unprecedented, and adds valuable insight into research on the longitudinal outcomes of literacy intervention.

The prediction of responsiveness to literacy intervention is paramount in mitigating adverse life outcomes associated with poor responsiveness. While the prediction outcomes typically predicate on binary profiles of responders and non-responders^38,39^, such classification may miss finer-grained heterogeneity. The clustering of predictor importance to identify responder and non-responder subgroup profiles represents a new attempt at accommodating the population heterogeneity.

Subgroup profiles promote the personalization of intervention to better address the learning characteristics of the students from different subgroups. More importantly, the prediction and clustering of subgroups based on broader baseline cognitive and achievement predictors – extending beyond measures like phonemic awareness that are traditionally specific to the assessment of dyslexia – is significant. A predictive model on intervention responsiveness that is trained on broader psychological data allows for more inclusive detection of at-risk learners across a diverse special needs population, regardless of the demonstration of conventional dyslexia symptomology or presence of co-morbidities.

Clustering via machine learning also demonstrated the predictive potential for the longitudinal outcomes of responsiveness within the respective subgroups. The inclusion of longitudinal data allows for more insightful findings – a perennial problem in dyslexia research, which is dominated by research on short-term outcomes^19^. Notably, subgroup membership was able to demonstrate the predictive potential of longitudinal outcomes by accommodating the dynamic, possibly non-linear profiles of students with learning difficulties not only during the intervention phase but years after intervention. Machine learning can detect signals that predict the trajectory of long-term literacy outcomes. Such detection of subtle signals means that even a child that performs ‘well’ at baseline testing needs to be monitored to detect any literacy issues over the long term. The clustering of subgroups that can predict longitudinal outcomes is highly beneficial for students as it provides better treatment response match, provide the most effective support that is effective over time and potentially reduce the risk of long-term academic failure among students who are predicted to be non-responders.

## Methods

### Dataset

A retrospective dataset of cognitive and achievement scores was collected from 849 students with diagnosed neurodevelopmental disorders (e.g., dyslexia, autism) who are attending mainstream primary or special education schools in Singapore^14^. The mean age of the students was 6.9 years (83.89 months) at baseline. The dataset consists of 196 baseline predictors that are largely pairwise differences of the continuous predictors that were created based on 23 baseline predictors - from normed psychological assessments - of the following categories: (1) background profile (e.g., gender, duration of early intervention), (2) nonverbal reasoning, (3) processing speed and working memory, (4) memory recall, (5) verbal comprehension, and (6) literacy (e.g., reading, spelling).

The dataset for this study is the dyslexia subset of 132 participants with long-term intervention data from a larger set of 849 students (the others only had baseline data with no outcomes data). The students in the dyslexia subset were diagnosed with dyslexia and received literacy intervention presented pre- (baseline) and immediate post-literacy intervention assessment scores, corresponding to time points 0 (T0) and 1 (T1), respectively. The mean age of the students was 8.2 years at baseline (98.08 months), with an age range of 6.2 years (74 months) to 12 years (144 months). A diagnosis of dyslexia is determined by a psychologist/pediatrician using one or more of the following sources: normed cognitive tests (e.g., WIAT), achievement tests (e.g., WRAT), and school records. The students were identified for diagnosis after demonstrating persistent literacy difficulties and evidence of underlying phonological processing deficits. In addition, the student must demonstrate a discrepancy between the general cognitive abilities and literacy performance, alongside significantly below-average scores on standardized reading and spelling tests (more than 1 standard deviation below the mean). Based on the DSM-5 TR^40^, students whose literacy difficulties were explained by intellectual disability or sensory impairments were excluded from the diagnosis of dyslexia.

These participants from the dyslexia subset received literacy intervention for an average of 33.62 months, with a range from 5 months to 115 months. The intervention program was based on the Orton-Gillingham (OG) approach or programs that are similarly aligned with the OG approach. These programs include the Essential Literacy Approach, Toe By Toe and Lindamood Bell. The OG approach is a prescriptive word-reading intervention for individuals with dyslexia that is based on the structured and sequential instruction of phonograms in a multisensorial manner^41,42^. The OG approach involves the introduction of phonograms, spelling rules, blending or syllabication techniques. A customized list of words including previously taught phonograms and a new phonogram concept is presented to the student for practice and reinforcement via a multisensory approach (i.e., visual, auditory, tactile, kinesthetic) to aid in concept acquisition. For instance, the phonogram *un* is shown on a flashcard with a picture of a person singing. The student sees the letters *un* with the visual, hears the sound /*un*/ after repeating the letters in the phonogram and the keyword ‘*sun*’, and traces the letters *un* on a rough surface. A curated word list including words like ‘gun’, ‘run’ and ‘fun’ is presented as practice words. Interspersed in the phonics curriculum is the introduction of morphological concepts like ‘suffix’, ‘prefix’ and ‘root’ to allow these individuals to understand the basis and formation of multisyllabic words.

Professionals like the Special Educational Needs Officers, literacy teachers and educational therapists administered the literacy intervention program. These professionals have undergone official training and certification in the administration of the program. In addition, these professionals have undergone further development in the understanding of various special needs profiles, including dyslexia, which cumulates into diplomas, post-graduate certificates or masters in domains related to special education. The intervention was implemented uniformly across participating schools, and the institutional context (e.g., similar curriculum and resources). Each session spanned one to two hours in a class of one to four students, with the total weekly dosage averaging over a span of three hours. The professionals were also expected to differentiate in classes that had varied profiles of learning styles and speed. Progression along the structured curriculum involves a mix of schedule-based and mastery-based progression that is dependent on the judgment of the professionals.

Binary outcome labels of ‘intervention responders’ and ‘intervention non-responders’ were determined previously using the dyslexia subset as ground truth via the discrepancy of T0 and T1 literacy scores, given that dyslexia is fundamentally a difficulty in reading and spelling^14^. Specifically, student progress was based on within-individual gains from baseline, in recognition of the variability in learning profiles among students with special educational needs. A data-driven clustering approach was adopted to establish class membership. A two-dimensional scatterplot was created using the literacy gain scores (reading and spelling difference scores). K-Means clustering, an unsupervised machine learning algorithm, was employed to systematically determine group membership for data points that were clustered near the zero-change boundary. Based on the Davies–Bouldin Index and visual inspection, a two-cluster solution was selected. Students in the cluster with higher average gains were labeled as responders while those in the cluster with lower or no gains were labeled as non-responders. 65 responders (43 males, 22 females) and 67 non-responders (48 males, 19 females) were identified. The mean age of the responders was 7.6 years at baseline (91.59 months), with an age range of 6.2 years (74 months) to 11.7 years (140 months). The mean age of the non-responders was 8.2 years at baseline (98.08 months), with an age range of 6.2 years (74 months) to 11.6 years (139 months). The percentage of non-responders was 50.75% in the dataset, which is highly congruent with previous estimates of literacy non-responders^11^. Semi-supervised learning models were trained and tested on the entire dataset. The top 20 LIME predictors of both responder and non-responder groups were generated based on the best-performing Gaussian Naïve Bayes model (F1 = 0.72). More information about the dataset and the definition of responder/non-responder categories can be found in this study^14^.

### Clustering procedure

In this study for research objective 1, K-Means clustering was implemented on the LIME coefficient values of dyslexia subset (derived from a local linear regression surrogate model) to further identify subgroups within the responder (*n* = 68) and non-responder groups (*n* = 64). The high-dimensional responder and non-responder datasets were dimensionally reduced to two dimensions using t-distributed Stochastic Neighbor Embedding (t-SNE). The number of subgroups per group was selected via the number of clusters (k) with the lowest Davies–Bouldin Index (DBI) score across all possible values presented. Lowest DBI represents within-cluster variation that is the smallest compared to between-cluster separation, i.e., the clusters being most distinct overall. The selection of the ideal number of clusters was also further assessed by visual inspection. Figures 1 and 2 show the clustering of responder and non-responder subgroups. Membership and the associated predictor values of each cluster were collated into smaller datasets. The values for each predictor were averaged per cluster. The predictors were ranked according to descending order of predictive contribution in each cluster. The mean discrepancy score for each predictor was compared against the global average of to be labeled either ‘high’ or ‘low’. Supplementary Table 1 shows the global average of top LIME predictors.

### Participant recruitment for longitudinal outcomes

To construct the dataset for longitudinal outcomes per subgroup for research objective 2, participants - from the dyslexia subset of 132 participants - who met the following requirements were recruited: Received at least 33 months of intervention (T0–T1) and graduated from intervention for at least 24 months (T1–T2). Applying these exclusionary criteria reduced the eligible pool from 132 to 82 participants. Due to the retrospective nature of recruitment and the extended follow-up interval, recruitment at T2 was constrained by practical factors. Of the 82 eligible participants, 41 participants did not agree to participate during the time of recall due to a variety of reasons (e.g., no longer residing in the country, military service, declined participation). 41 participants were recruited. Recruitment was conducted without prior knowledge of responder status or subgroup membership, in order to avoid researcher selection bias. Consequently, the recruitment of participants achieved approximately equal group sizes. Recruited participants from the responding (*n* = 20) and non-responding (*n* = 21) groups underwent follow-up testing (T2). The average intervention duration (T0–T1) was 71.71 months, while follow-up duration (T1–T2) was 68.2 months.

The exclusionary criteria were implemented to observe much stricter standards as compared to the prevailing benchmarks. The mean duration of 71.71 months of intervention in our study was much longer than benchmarks of 4.84 months^19^ and 24 months^18^. The mean follow-up duration (T1–T2) was 68.2 months, which represented more than five years of post-intervention. This mean follow-up duration of this study is more than 5 times the average follow-up duration from the meta-analysis that did not identify a study with a follow-up of more than 4 years (48 months) among the 70 dyslexia intervention papers that were reviewed^19^. The emphasis on a long follow-up period was intentional. While a shorter follow-up interval could have increased the achievable sample size, it would have weakened the study’s ability to address the documented gap in very long-term outcomes of dyslexia intervention. Nonetheless, we formally tested whether the participants retained for T2 were similar to the non-participating T1 students. First, participants retained in T2 did not differ significantly from the non-participants (*p* > 0.05). Second, we ran a Chi-square test comparing the distribution across the subgroups between T1 and T2, finding non-statistical significance (χ²(5) = 7.45, *p* = 0.19). This suggests that the T2 subgroup membership proportions are very similar to the baseline T1 subgroup membership proportions, lending confidence that non-participation did not unduly affect subgroup membership distribution.

### Measures

The participants were tested on four normed achievement tasks from the Wechsler Individual Achievement Test^43^: Word Reading, Word Spelling, Pseudoword Decoding and Reading Comprehension.

### Human ethics and consent to participate

This study was conducted in accordance with the ethical principles outlined in the Declaration of Helsinki. Ethical approval for this study was obtained from the Nanyang Technological University Institutional Review Board (IRB-2024-241). All participants provided informed consent prior to their inclusion in the study. The purpose and procedures of the research were clearly explained to each participant, and they were informed of their right to withdraw from the study at any time without any negative consequences. Written consent was obtained in accordance with ethical guidelines and IRB approval.

### Procedure

The participants were grouped according to the subgroups that were predicted and clustered by the Gaussian Naïve Bayes model and K-Means, respectively. The standard scores of these four tasks were plotted across three time points as a function of duration.

## Supplementary information

Supplementary information.

## Data Availability

The primary and reference datasets are available from the authors upon reasonable request. The datasets generated and/or analyzed during the current study are not publicly available because they contain university-owned data and information on students with special educational needs, a vulnerable population, which cannot be openly shared. Data is available from the corresponding author on reasonable request and with appropriate permissions.

## Code availability

Code is available from the authors upon reasonable request. There are no restrictions to access.

## References

[1] Boston Consulting Group, & UCSF Dyslexia Center. *The Economic Impact of Dyslexia on California [White paper]*, (Boston Consulting Group, & UCSF Dyslexia Center, 2020).

[2] Coughlin, S. S., Vernon, M., Hatzigeorgiou, C. & George, V. Health literacy, social determinants of health, and disease prevention and control. *J. Environ. Health Sci.* **6**, 3061 (2020). 33604453 PMC7889072

[3] Brucki, S. M. D. Illiteracy and dementia. *Dement. Neuropsychol.* **4**, 153–157 (2010). 29213680 10.1590/S1980-57642010DN40300002 PMC5619283

[4] DeWalt, D. A., Berkman, N. D., Sheridan, S., Lohr, K. N. & Pignone, M. P. Literacy and health outcomes: a systematic review of the literature. *J. Gen. Intern. Med.* **19**, 1228–1239 (2004). 15610334 10.1111/j.1525-1497.2004.40153.x PMC1492599

[5] Stanovich, K. E. Explaining the differences between the dyslexic and the garden-variety poor reader: the phonological-core variable-difference model. *J. Learn Disabil.* **21**, 590–604 (1988). 2465364 10.1177/002221948802101003

[6] Wolf, M. & Bowers, P. G. The double-deficit hypothesis for the developmental dyslexias. *J. Educ. Psychol.* **91**, 415–438 (1999).

[7] Stein, J. The magnocellular theory of developmental dyslexia. *Dyslexia* **7**, 12–36 (2001). 11305228 10.1002/dys.186

[8] Nicolson, R. I., Fawcett, A. J. & Dean, P. Developmental dyslexia: the cerebellar deficit hypothesis. *Trends Neurosci.* **24**, 508–511 (2001). 11506881 10.1016/s0166-2236(00)01896-8

[9] Thomas, P.L. *The Science of Reading Movement: The Never-Ending Debate and the Need for a Different Approach to Reading Instruction*. 36 (Boulder, CO: National Education Policy Center, 2022).

[10] Johnston, P. & Scanlon, D. An examination of dyslexia research and instruction with policy implications. *Lit. Res.* **70**, 107–128 (2021).

[11] Al Otaiba, S. & Fuchs, D. Characteristics of children who are unresponsive to early literacy intervention: a review of the literature. *Remedial Spec. Educ.* **23**, 300–316 (2002).

[12] Al Otaiba, S. & Fuchs, D. Who are the young children for whom best practices in reading are ineffective?: An experimental and longitudinal study. *J. Learn Disabil.* **39**, 414–431 (2006). 17004674 10.1177/00222194060390050401

[13] Blockmans, L., Kievit, R., Wouters, J., Ghesquière, P. & Vandermosten, M. Dynamics of cognitive predictors during reading acquisition in a sample of children overrepresented for dyslexia risk. *Dev. Sci.* **27**, e13412 (2024). 37219071 10.1111/desc.13412

[14] Tan, A. S.-C., Ali, F., Poon, K. & Lee, C. L. Predicting Literacy Intervention Responsiveness Using Semi-Supervised Machine Learning. *Res. Dev. Disabil.* **165**, 105090 (2025). 10.1016/j.ridd.2025.105090 40845520

[15] Tilanus, E. A. T., Segers, E. & Verhoeven, L. Predicting responsiveness to a sustained reading and spelling intervention in children with dyslexia. *Dyslexia* **25**, 190–206 (2019). 31016832 10.1002/dys.1614 PMC6593814

[16] Van Der Kleij, S. W., Segers, E., Groen, M. A. & Verhoeven, L. Response to intervention as a predictor of long-term reading outcomes in children with dyslexia. *Dyslexia* **23**, 268–282 (2017). 28691254 10.1002/dys.1562

[17] Sanders, E. A., Berninger, V. W. & Abbott, R. D. Sequential prediction of literacy achievement for specific learning disabilities contrasting in impaired levels of language in grades 4 to 9. *J. Learn Disabil.* **51**, 137–157 (2018). 28199175 10.1177/0022219417691048 PMC5538955

[18] Middleton, A. E., Farris, E. A., Ring, J. J. & Odegard, T. N. Predicting and evaluating treatment response: evidence toward protracted response patterns for severely impacted students with dyslexia. *J. Learn Disabil.* **55**, 272–291 (2022). 34612740 10.1177/00222194211047633

[19] Suggate, S. P. A meta-analysis of the long-term effects of phonemic awareness, phonics, fluency, and reading comprehension interventions. *J. Learn Disabil.* **49**, 77–96 (2016). 24704662 10.1177/0022219414528540

[20] Ribeiro, M. T., Singh, S. & Guestrin, C. ‘Why should i trust you?’ Explaining the predictions of any classifier. In *Proceedings of the 22nd ACM SIGKDD international conference on knowledge discovery and data mining* 1135–1144 (ACM, 2016).

[21] Astle, D. E., Holmes, J., Kievit, R. & Gathercole, S. E. Annual Research Review: The transdiagnostic revolution in neurodevelopmental disorders. *J. Child Psychol. Psychiatry* **63**, 397–417 (2022). 34296774 10.1111/jcpp.13481

[22] Tan, A. S., Ali, F. & Poon, K. K. Subjective well-being of children with special educational needs: Longitudinal predictors using machine learning. *Appl. Psych Health Well* **17**, e12625 (2025). 10.1111/aphw.12625 39529312

[23] Song, S., Zhang, Y., Shu, H., Su, M. & McBride, C. Universal and specific predictors of Chinese children with dyslexia – exploring the cognitive deficits and subtypes. *Front. Psychol.* **10**, 2904 (2020). 31969853 10.3389/fpsyg.2019.02904 PMC6960230

[24] Goh, P. K., Lee, C. A., Martel, M. M., Karalunas, S. L. & Nigg, J. T. Subgroups of childhood ADHD based on temperament traits and cognition: concurrent and predictive validity. *J. Abnorm. Child Psychol.* **48**, 1251–1264 (2020). 32666315 10.1007/s10802-020-00668-x PMC8647195

[25] Mandelli, V. et al. Prognostic early snapshot stratification of autism based on adaptive functioning. *Nat. Ment. Health* **1**, 327–336 (2023).

[26] Johnson, S. et al. Learning disabilities among extremely preterm children without neurosensory impairment: Comorbidity, neuropsychological profiles and scholastic outcomes. *Early Hum. Dev.* **103**, 69–75 (2016). 27517525 10.1016/j.earlhumdev.2016.07.009

[27] Eide, M. G., Øyen, N., Skjærven, R. & Bjerkedal, T. Associations of birth size, gestational age, and adult size with intellectual performance: evidence from a cohort of Norwegian men. *Pediatr. Res.* **62**, 636–642 (2007). 17805203 10.1203/PDR.0b013e31815586e9

[28] Yin, W. et al. Gestational age and risk of intellectual disability: a population-based cohort study. *Arch. Dis. Child* **107**, 826–832 (2022). 35470219 10.1136/archdischild-2021-323308 PMC9411878

[29] Heuvelman, H. et al. Gestational age at birth and risk of intellectual disability without a common genetic cause. *Eur. J. Epidemiol.* **33**, 667–678 (2018). 29214412 10.1007/s10654-017-0340-1 PMC6061122

[30] Gleason, J. L. et al. Gestational age at term delivery and children’s neurocognitive development. *Int. J. Epidemiol.* **50**, 1814–1823 (2022). 34999875 10.1093/ije/dyab134 PMC8932293

[31] Nielsen, T. M., Pedersen, M. V., Milidou, I., Glavind, J. & Henriksen, T. B. Long-term cognition and behavior in children born at early term gestation: a systematic review. *Acta Obstet. Gynecol. Scand.* **98**, 1227–1234 (2019). 31091336 10.1111/aogs.13644

[32] Nivins, S., Padilla, N., Kvanta, H. & Ådén, U. Gestational age and cognitive development in childhood. *JAMA Netw. Open* **8**, e254580 (2025). 40227687 10.1001/jamanetworkopen.2025.4580 PMC11997729

[33] MacKay, D. F., Smith, G. C. S., Dobbie, R. & Pell, J. P. Gestational age at delivery and special educational need: retrospective cohort study of 407,503 schoolchildren. *PLoS Med.* **7**, e1000289 (2010). 20543995 10.1371/journal.pmed.1000289 PMC2882432

[34] Berninger, V. W. & Abbott, R. D. Differences between children with dyslexia who are and are not gifted in verbal reasoning. *Gifted Child Q.* **57**, 223–233 (2013). 10.1177/0016986213500342 PMC3829472 24249873

[35] Holmes, V. M. & Quinn, L. Unexpectedly poor spelling and phonological-processing skill. *Sci. Stud. Read.* **13**, 295–317 (2009).

[36] Holmes, V. M., Malone, A. M. & Redenbach, H. Orthographic processing and visual sequential memory in unexpectedly poor spellers. *J. Res. Read.* **31**, 136–156 (2008).

[37] Kolinsky, R. & Tossonian, M. Phonological and orthographic processing in basic literacy adults and dyslexic children. *Read. Writ.* **36**, 1705–1742 (2023). 10.1007/s11145-022-10347-6 PMC9476393 36124227

[38] O Brien, B. A. et al. Response and non-response to intervention for reading difficulties: what role do cognitive correlates play?. *APJDD* **10**, 187–210 (2023).

[39] Aboud, K. S., Barquero, L. A. & Cutting, L. E. Prefrontal mediation of the reading network predicts intervention response in dyslexia. *Cortex* **101**, 96–106 (2018). 29459284 10.1016/j.cortex.2018.01.009 PMC5869156

[40] American Psychiatric Association, [APA]. Diagnostic and statistical manual of mental disorders 5. (American Psychiatric Association, 2013).

[41] Orton-Gillingham Academy. *What is the Orton-Gillingham approach?* https://www.ortonacademy.org/resources/what-is-the-orton-gillingham-approach/ (2020).

[42] Ring, J. J., Avrit, K. J. & Black, J. L. Take flight: the evolution of an Orton Gillingham-based curriculum. *Ann. Dyslexia* **67**, 383–400 (2017). 29134479 10.1007/s11881-017-0151-9

[43] Wechsler, D. *Wechsler Individual Achievement Test–Fourth Edition (WIAT–4*) (Pearson, 2018).

