# Supplementary material for: Uncovering subgroup heterogeneity in dyslexia intervention outcomes using explainable machine learning
Source: NPJ Sci Learn. 2026 May 25;11:47. doi: 10.1038/s41539-026-00429-3 (PMC13482360; doi:10.1038/s41539-026-00429-3)
Supplement: Supplementary file 1 [file 41539_2026_429_MOESM1_ESM.pdf]

Supplementary Information for Uncovering Subgroup Heterogeneity in Dyslexia Intervention Outcomes Using Explainable Machine Learning

Supplementary Table 1: Overall Average of Top LIME predictors

LIME Feature / Predictor	Overall Average Score or Value
Gestation (months)	39.18
Duration of Early Intervention (months)	10.32
Verbal Comprehension: Similarities	89.66
Memory: Recall of Picture	93.83
Memory: Recall of Object (Immediate Average)	97.66
Memory: Recall of Object (Immediate Spatial)	95.35
Memory: Recall of Object (Delayed Verbal) - Memory: Recall of Object (Delayed Average)	0.42
Memory: Recall of Object (Immediate Average) - Verbal Comprehension: Similarities	8.00
Memory: Recall of Object (Immediate Verbal) - Verbal Comprehension: Similarities	5.69
Nonverbal: Sequential and Quantitative Reasoning - Memory: Recall of Object (IS)	2.16
Digit Span: Average - Memory: Recall of Object (IS)	-1.42

Supplementary Table 2: LIME Feature Interpretation Table

Feature / Predictor	LIME Contribution	Mean Discrepancy Score	Interpretation
**Responder - Subgroup 1**			
Gestation (months)	Negative (-)	39.84 (High)	High score contributed negatively to the predicted probability of the responder group.
Memory: Recall of Picture	Negative (-)	97.91 (High)	High score contributed negatively to the predicted probability of the responder group.
Duration of Early Intervention (months)	Positive (+)	4.37 (Low)	Low score contributed positively to the predicted probability of the responder group.
Memory: Recall of Object (Immediate-Average)	Negative (-)	96.76 (Low)	Low score contributed negatively to the predicted probability of the responder group.
Verbal Comprehension: Similarities	Positive (+)	98.74 (High)	High score contributed positively to the predicted probability of the responder group.
**Responder - Subgroup 2**			
Gestation (months)	Positive (+)	35.24 (Low)	Low score contributed positively to the predicted probability of the responder group.
Memory: Recall of Object (Immediate Spatial)	Positive (+)	84.76 (Low)	Low score contributed positively to the predicted probability of the responder group.
Memory: Recall of Object (Immediate Average)	Positive (+)	81.83 (Low)	Low score contributed positively to the predicted probability of the responder group.
Memory: Recall of Object (Delayed Verbal) - Memory: Recall of Object (Delayed Average)	Positive (+)	1.28 (High)	High score contributed positively to the predicted probability of the responder group.
Verbal Comprehension: Similarities	Negative (-)	89.57 (Low)	Low score contributed negatively to the predicted probability of the responder group.
**Non-responder - Subgroup 1**			
Gestation (months)	Positive (+)	40 (High)	High score contributed positively to the predicted probability of the non-responder group.
Memory: Recall of Picture	Positive (+)	100.88 (High)	High score contributed positively to the predicted probability of the non-responder group.
Duration of Early Intervention (months)	Negative (-)	3.70 (Low)	Low score contributed negatively to the predicted probability of the non-responder group.
Memory: Recall of Object (Immediate Average) - Verbal Comprehension: Similarities	Positive (+)	20.62 (High)	High score contributed positively to the predicted probability of the non-responder group.
Memory: Recall of Object (Immediate Verbal) - Verbal Comprehension: Similarities	Positive (+)	8.54 (High)	High score contributed positively to the predicted probability of the non-responder group.
**Non-responder - Subgroup 2**			
Gestation (months)	Positive (+)	40.06 (High)	High score contributed positively to the predicted probability of the non-responder group.
Memory: Recall of Object (Immediate Spatial)	Positive (+)	110.27 (High)	High score contributed positively to the predicted probability of the non-responder group.
Memory: Recall of Object (Immediate Average)	Positive (+)	97.86 (High)	High score contributed positively to the predicted probability of the non-responder group.
Memory: Recall of Object (Delayed Verbal) - Memory: Recall of Object (Delayed Average)	Positive (+)	-7.80 (Low)	Low score contributed positively to the predicted probability of the non-responder group.

Verbal Comprehension: Similarities	Negative (-)	89.72 (High)	High score contributed negatively to the predicted probability of the non-responder group.
**Non-responder - Subgroup 3**			
Gestation (months)	Positive (+)	39.94 (High)	High score contributed positively to the predicted probability of the non-responder group.
Memory: Recall of Picture	Positive (+)	92.65 (Low)	Low score contributed positively to the predicted probability of the non-responder group.
Memory: Recall of Object (Immediate Average) - Verbal Comprehension: Similarities	Positive (+)	13.78 (High)	High score contributed positively to the predicted probability of the non-responder group.
Duration of Tier 1 Early intervention (months)	Positive (+)	20.25 (High)	High score contributed positively to the predicted probability of the non-responder group.
Memory: Recall of Object (Immediate Verbal) - Verbal Comprehension: Similarities	Positive (+)	15.70 (High)	High score contributed positively to the predicted probability of the non-responder group.
**Non-responder - Subgroup 4**			
Gestation (months)	Negative (-)	37.14 (Low)	Low score contributed negatively to the predicted probability of the non-responder group.
Duration of Early Intervention (months)	Positive (+)	32.57 (High)	High score contributed positively to the predicted probability of the non-responder group.
Nonverbal: Sequential and Quantitative Reasoning - Memory: Recall of Object (IS)	Positive (+)	-14.43 (Low)	Low score contributed positively to the predicted probability of the non-responder group.
Digit Span: Average - Memory: Recall of Object (IS)	Positive (+)	-19.93 (Low)	Low score contributed positively to the predicted probability of the non-responder group.
Memory: Recall of Picture	Positive (+)	90.14 (Low)	Low score contributed positively to the predicted probability of the non-responder group.

High = Higher than overall average
Low = Lower than overall average

Supplementary Table 3: Average Metrics or Scores per Responder Cluster at Baseline (T0)

Metrics	Subgroup 1 (n = 48)	Subgroup 2 (n = 17)
Gestation (weeks)	39.85 (0.07)	35.24 (0.58)
Duration of Tier 1 Early Intervention (months)	4.37 (1.51)	21.94 (4.29)
Duration of Tier 3 Intervention (months)	3.37 (1.11)	4.18 (1.41)
Word Reading	91.26 (1.85)	82.61 (3.60)
Word Spelling	92.39 (1.75)	81.59 (4.12)
Memory: Recall of Picture	97.91 (2.28)	87.70 (3.70)
Memory: Recall of Object (IV)	101.58 (1.82)	88.00 (3.77)
Memory: Recall of Object (IS)	92.67 (1.58)	84.76 (3.06)
Memory: Recall of Object (IA)	96.76 (1.46)	86.38 (2.99)
Memory: Recall of Object (DV)	99.20 (1.89)	88.41 (3.37)
Memory: Recall of Object (DS)	93.30 (1.56)	85.84 (3.37)
Memory: Recall of Object (DA)	96.37 (1.50)	87.12 (2.99)
Verbal Comprehension: Word Definitions	96.72 (1.93)	91.73 (3.62)
Verbal Comprehension: Similarities	98.74 (1.76)	89.57 (3.09)
Nonverbal: Matrix	103.62 (1.88)	80.37 (3.90)
Nonverbal: Sequential and Quantitative Reasoning	105.41 (1.80)	84.68 (3.41)
Spatial: Pattern of Construction	102.95 (1.72)	88.88 (3.61)
Spatial: Recall of Design	103.04 (1.78)	88.00 (4.28)
Speed of Information Processing	106.80 (3.12)	93.24 (3.75)
Digit Span: Recall of Digit Forward	111.52 (3.40)	83.00 (3.26)
Digit Span: Recall of Digit Backwards	94.00 (1.96)	81.83 (2.52)
Digit Span: Average	102.77 (1.91)	82.41 (2.14)

Supplementary Table 4: Average Metrics or Scores per Non-responder Cluster at Baseline (T0)

	**Non-responders**			
Metrics	Subgroup 1 (n=27)	Subgroup 2 (n=18)	Subgroup 3 (n = 15)	Subgroup 4 (n=7)
Gestation (weeks)	40 .00 (0.00)	40.06 (0.06)	39.94 (0.06)	37.14 (0.26)
Duration of Tier 1 early intervention (months)	3.70 (1.53)	7.00 (2.75)	20.25 (4.36)	32.57 (4.31)
Duration of Tier 3 Intervention (months)	7.63 (2.02)	10.17 (2.49)	5.44 (2.39)	8.57 (4.31)
Word Reading	80.06 (1.95)	81.44 (2.16)	80.73 (3.23)	83.86 (6.11)
Word Spelling	84.02 (2.03)	85.11 (2.07)	82.29 (2.85)	80.71 (5.55)
Memory: Recall of Picture	100.88 (2.24)	81.07 (4.28)	92.65 (3.09)	90.14 (4.70)
Memory: Recall of Object (IV)	108.51 (4.21)	97.86 (2.95)	96.06 (2.76)	99.29 (7.02)
Memory: Recall of Object (IS)	96.00 (1.57)	110.27 (2.58)	94.98 (2.29)	98.71 (5.51)
Memory: Recall of Object (IA)	101.97 (2.58)	106.79 (1.84)	94.14 (2.38)	99.00 (5.42)
Memory: Recall of Object (DV)	107.86 (2.91)	102.52 (2.91)	81.83 (6.48)	104.43 (5.45)
Memory: Recall of Object (DS)	96.88 (1.13)	111.83 (2.25)	93.66 (1.46)	102.29 (4.41)
Memory: Recall of Object (DA)	102.55 (1.79)	110.32 (2.98)	88.52 (3.66)	103.36 (4.09)
Verbal Comprehension: Word Definitions	74.58 (2.27)	100.89 (3.37)	79.32 (3.00)	91.57 (6.30)
Verbal Comprehension: Similarities	81.35 (3.06)	85.72 (2.70)	80.36 (3.32)	83.43 (6.75)
Nonverbal: Matrix	100.39 (1.79)	89.72 (3.82)	97.31 (2.75)	82.00 (7.83)
Nonverbal: Sequential and Quantitative	97.42 (1.48)	97.04 (2.13)	94.91 (3.04)	84.29 (6.72)
Reasoning	104.05 (1.69)	105.11 (3.62)	101.66 (2.57)	95.29 (6.82)
Spatial: Pattern of Construction	103.63 (2.72)	106.74 (2.30)	101.19 (2.84)	93.14 (6.33)
Spatial: Recall of Design	107.41 (2.64)	100.72 (2.67)	94.76 (6.27)	94.57 (7.02)
Speed of Information Processing	88.52 (2.99)	97.74 (3.81)	96.00 (4.57)	79.86 (5.00)
Digit Span: Recall of Digit Forward	91.84 (2.38)	93.72 (1.99)	87.78 (2.32)	77.71 (5.05)
Digit Span: Recall of Digit Backwards	90.07 (2.01)	95.72 (2.32)	91.89 (3.19)	78.79 (4.59)
Digit Span: Average				

Supplementary Table 5: Mann Whitney Test Comparing the Longitudinal Outcomes of Subgroups (p-values adjusted for multiple comparisons using FDR)

T1-T0 Reading

	R (SG1)	NR (SG1)	NR (SG2)	NR (SG3)
R (SG1)	-	-	-	-
NR (SG1)	< .001*	-	-	-
NR (SG2)	0.036*	0.893	-	-
NR (SG3)	0.009*	0.639	0.754	-

T2-T1 Reading

	R (SG1)	NR (SG1)	NR (SG2)	NR (SG3)
R (SG1)	-	-	-	-
NR (SG1)	0.566	-	-	-
NR (SG2)	0.651	0.566	-	-
NR (SG3)	0.050*	0.022*	0.566	-

T1-T0 Spelling

	R (SG1)	NR (SG1)	NR (SG2)	NR (SG3)
R (SG1)	-	-	-	-
NR (SG1)	< .001*	-	-	-
NR (SG2)	0.156	0.864	-	-
NR (SG3)	0.009*	0.864	0.864	-

T2-T1 Spelling

	R (SG1)	NR (SG1)	NR (SG2)	NR (SG3)
R (SG1)	-	-	-	-
NR (SG1)	0.355	-	-	-
NR (SG2)	0.840	0.651	-	-
NR (SG3)	0.229	0.120	0.229	-

T1-T0 Pseudoword Decoding

	R (SG1)	NR (SG1)	NR (SG2)	NR (SG3)
R (SG1)	-	-	-	-
NR (SG1)	< .001*	-	-	-
NR (SG2)	0.023*	0.073	-	-
NR (SG3)	0.864	0.120	0.086	-

T2-T1 Pseudoword Decoding

	R (SG1)	NR (SG1)	NR (SG2)	NR (SG3)
R (SG1)	-	-	-	-
NR (SG1)	0.188	-	-	-
NR (SG2)	0.229	0.188	-	-
NR (SG3)	0.187	0.703	0.187	-

T1-T0 Reading Comprehension

	R (SG1)	NR (SG1)	NR (SG2)	NR (SG3)
R (SG1)	-	-	-	-
NR (SG1)	0.271	-	-	-
NR (SG2)	0.271	0.857	-	-
NR (SG3)	0.271	0.857	0.857	-

T2-T1 Reading Comprehension

	R (SG1)	NR (SG1)	NR (SG2)	NR (SG3)
R (SG1)	-	-	-	-
NR (SG1)	0.831	-	-	-
NR (SG2)	0.797	0.798	-	-
NR (SG3)	0.831	0.798	0.798	-

R = Responders | NR = Non-responders

Supplementary Table 6: Effect Sizes Corresponding to Mann-Whitney Tests for Longitudinal Outcomes of Subgroups

T1-T0 Reading

	R (SG1)	NR (SG1)	NR (SG2)	NR (SG3)
R (SG1)	-	-	-	-
NR (SG1)	0.748	-	-	-
NR (SG2)	0.526	0.050	-	-
NR (SG3)	0.644	0.206	0.267	-

T2-T1 Reading

	R (SG1)	NR (SG1)	NR (SG2)	NR (SG3)
R (SG1)	-	-	-	-
NR (SG1)	0.144	-	-	-
NR (SG2)	0.110	0.252	-	-
NR (SG3)	0.499	0.714	0.334	-

T1-T0 Spelling

	R (SG1)	NR (SG1)	NR (SG2)	NR (SG3)
R (SG1)	-	-	-	-
NR (SG1)	0.784	-	-	-
NR (SG2)	0.395	0.118	-	-
NR (SG3)	0.644	0.055	0.134	-

T2-T1 Spelling

	R (SG1)	NR (SG1)	NR (SG2)	NR (SG3)
R (SG1)	-	-	-	-
NR (SG1)	0.216	-	-	-
NR (SG2)	0.0548	0.168	-	-
NR (SG3)	0.3629	0.577	0.668	-

T1-T0 Pseudoword Decoding

	R (SG1)	NR (SG1)	NR (SG2)	NR (SG3)
R (SG1)	-	-	-	-
NR (SG1)	0.633	-	-	-
NR (SG2)	0.592	0.538	-	-
NR (SG3)	0.045	0.412	0.802	-

T2-T1 Pseudoword Decoding

	R (SG1)	NR (SG1)	NR (SG2)	NR (SG3)
R (SG1)	-	-	-	-
NR (SG1)	0.312764	-	-	-
NR (SG2)	0.296078	0.437237	-	-
NR (SG3)	0.335754	0.109847	0.668153	-

T1-T0 Reading Comprehension

	R (SG1)	NR (SG1)	NR (SG2)	NR (SG3)
R (SG1)	-	-	-	-
NR (SG1)	0.291	-	-	-
NR (SG2)	0.395	0.151	-	-
NR (SG3)	0.327	0.069	0.134	-

T2-T1 Reading Comprehension

	R (SG1)	NR (SG1)	NR (SG2)	NR (SG3)
R (SG1)	-	-	-	-
NR (SG1)	0.058	-	-	-
NR (SG2)	0.219	0.370	-	-
NR (SG3)	0.054	0.165	0.401	-

Note. Effect sizes (r) were derived from Mann–Whitney U test statistics.

R = Responders | NR = Non-responders

Supplementary Table 7: Complete List of Predictors of Intervention Responsiveness in the Dataset

Duration of Tier 1 Early intervention (months)
Duration of Tier 3 Intervention (months)
Nonverbal: Matrix
Nonverbal: Sequential and Quantitative Reasoning
Spatial: Pattern of Construction
Spatial: Recall of Design
Speed of Information Processing
Digit Span: Recall of Digit Forward
Digit Span: Recall of Digit Backwards
Digit Span: Average
Memory: Recall of Picture
Memory: Recall of Object (IV)
Memory: Recall of Object (IS)
Memory: Recall of Object (IA)
Memory: Recall of Object (DV)
Memory: Recall of Object (DS)
Memory: Recall of Object (DA)
Verbal Comprehension: Word Definitions
Verbal Comprehension: Similarities
Single Word Reading (T0)
Single Word Spelling (T0)
Single Word Reading (T0) - Single Word Spelling (T0)
Duration of Tier 1 Early intervention - Duration of Tier 3 Intervention
Nonverbal: Matrix - Nonverbal: Sequential and Quantitative Reasoning
Nonverbal: Matrix - Spatial: Pattern of Construction
Nonverbal: Matrix - Spatial: Recall of Design
Nonverbal: Matrix - Speed of Information Processing
Nonverbal: Matrix - Digit Span: Recall of Digit Forward
Nonverbal: Matrix - Digit Span: Recall of Digit Backwards
Nonverbal: Matrix - Digit Span: Average
Nonverbal: Matrix - Memory: Recall of Picture
Nonverbal: Matrix - Memory: Recall of Object (IV)
Nonverbal: Matrix - Memory: Recall of Object (IS)
Nonverbal: Matrix - Memory: Recall of Object (IA)
Nonverbal: Matrix - Memory: Recall of Object (DV)
Nonverbal: Matrix - Memory: Recall of Object (DS)
Nonverbal: Matrix - Memory: Recall of Object (DA)
Nonverbal: Matrix - Verbal Comprehension: Word Definitions
Nonverbal: Matrix - Verbal Comprehension: Similarities
Nonverbal: Matrix - Single Word Reading (T0)
Nonverbal: Matrix - Single Word Spelling (T0)

Nonverbal: Sequential and Quantitative Reasoning - Spatial: Pattern of Construction
Nonverbal: Sequential and Quantitative Reasoning - Spatial: Recall of Design
Nonverbal: Sequential and Quantitative Reasoning - Speed of Information Processing
Nonverbal: Sequential and Quantitative Reasoning - Digit Span: Recall of Digit Forward
Nonverbal: Sequential and Quantitative Reasoning - Digit Span: Recall of Digit Backwards
Nonverbal: Sequential and Quantitative Reasoning - Digit Span: Average
Nonverbal: Sequential and Quantitative Reasoning - Memory: Recall of Picture
Nonverbal: Sequential and Quantitative Reasoning - Memory: Recall of Object (IV)
Nonverbal: Sequential and Quantitative Reasoning - Memory: Recall of Object (IS)
Nonverbal: Sequential and Quantitative Reasoning - Memory: Recall of Object (IA)
Nonverbal: Sequential and Quantitative Reasoning - Memory: Recall of Object (DV)
Nonverbal: Sequential and Quantitative Reasoning - Memory: Recall of Object (DS)
Nonverbal: Sequential and Quantitative Reasoning - Memory: Recall of Object (DA)
Nonverbal: Sequential and Quantitative Reasoning - Verbal Comprehension: Word Definitions
Nonverbal: Sequential and Quantitative Reasoning - Verbal Comprehension: Similarities
Nonverbal: Sequential and Quantitative Reasoning - Single Word Reading (T0)
Nonverbal: Sequential and Quantitative Reasoning - Single Word Spelling (T0)
Spatial: Pattern of Construction - Spatial: Recall of Design
Spatial: Pattern of Construction - Speed of Information Processing
Spatial: Pattern of Construction - Digit Span: Recall of Digit Forward
Spatial: Pattern of Construction - Digit Span: Recall of Digit Backwards
Spatial: Pattern of Construction - Digit Span: Average
Spatial: Pattern of Construction - Memory: Recall of Picture
Spatial: Pattern of Construction - Memory: Recall of Object (IV)
Spatial: Pattern of Construction - Memory: Recall of Object (IS)
Spatial: Pattern of Construction - Memory: Recall of Object (IA)
Spatial: Pattern of Construction - Memory: Recall of Object (DV)
Spatial: Pattern of Construction - Memory: Recall of Object (DS)
Spatial: Pattern of Construction - Memory: Recall of Object (DA)
Spatial: Pattern of Construction - Verbal Comprehension: Word Definitions
Spatial: Pattern of Construction - Verbal Comprehension: Similarities
Spatial: Pattern of Construction - Single Word Reading (T0)
Spatial: Pattern of Construction - Single Word Spelling (T0)
Spatial: Recall of Design - Speed of Information Processing
Spatial: Recall of Design - Digit Span: Recall of Digit Forward
Spatial: Recall of Design - Digit Span: Recall of Digit Backwards
Spatial: Recall of Design - Digit Span: Average
Spatial: Recall of Design - Memory: Recall of Picture
Spatial: Recall of Design - Memory: Recall of Object (IV)
Spatial: Recall of Design - Memory: Recall of Object (IS)
Spatial: Recall of Design - Memory: Recall of Object (IA)
Spatial: Recall of Design - Memory: Recall of Object (DV)
Spatial: Recall of Design - Memory: Recall of Object (DS)
Spatial: Recall of Design - Memory: Recall of Object (DA)

Spatial: Recall of Design - Verbal Comprehension: Word Definitions
Spatial: Recall of Design - Verbal Comprehension: Similarities
Spatial: Recall of Design - Single Word Reading (T0)
Spatial: Recall of Design - Single Word Spelling (T0)
Speed of Information Processing - Digit Span: Recall of Digit Forward
Speed of Information Processing - Digit Span: Recall of Digit Backwards
Speed of Information Processing - Digit Span: Average
Speed of Information Processing - Memory: Recall of Picture
Speed of Information Processing - Memory: Recall of Object (IV)
Speed of Information Processing - Memory: Recall of Object (IS)
Speed of Information Processing - Memory: Recall of Object (IA)
Speed of Information Processing - Memory: Recall of Object (DV)
Speed of Information Processing - Memory: Recall of Object (DS)
Speed of Information Processing - Memory: Recall of Object (DA)
Speed of Information Processing - Verbal Comprehension: Word Definitions
Speed of Information Processing - Verbal Comprehension: Similarities
Speed of Information Processing - Single Word Reading (T0)
Speed of Information Processing - Single Word Spelling (T0)
Digit Span: Recall of Digit Forward - Digit Span: Recall of Digit Backwards
Digit Span: Recall of Digit Forward - Digit Span: Average
Digit Span: Recall of Digit Forward - Memory: Recall of Picture
Digit Span: Recall of Digit Forward - Memory: Recall of Object (IV)
Digit Span: Recall of Digit Forward - Memory: Recall of Object (IS)
Digit Span: Recall of Digit Forward - Memory: Recall of Object (IA)
Digit Span: Recall of Digit Forward - Memory: Recall of Object (DV)
Digit Span: Recall of Digit Forward - Memory: Recall of Object (DS)
Digit Span: Recall of Digit Forward - Memory: Recall of Object (DA)
Digit Span: Recall of Digit Forward - Verbal Comprehension: Word Definitions
Digit Span: Recall of Digit Forward - Verbal Comprehension: Similarities
Digit Span: Recall of Digit Forward - Single Word Reading (T0)
Digit Span: Recall of Digit Forward - Single Word Spelling (T0)
Digit Span: Recall of Digit Backwards - Digit Span: Average
Digit Span: Recall of Digit Backwards - Memory: Recall of Picture
Digit Span: Recall of Digit Backwards - Memory: Recall of Object (IV)
Digit Span: Recall of Digit Backwards - Memory: Recall of Object (IS)
Digit Span: Recall of Digit Backwards - Memory: Recall of Object (IA)
Digit Span: Recall of Digit Backwards - Memory: Recall of Object (DV)
Digit Span: Recall of Digit Backwards - Memory: Recall of Object (DS)
Digit Span: Recall of Digit Backwards - Memory: Recall of Object (DA)
Digit Span: Recall of Digit Backwards - Verbal Comprehension: Word Definitions
Digit Span: Recall of Digit Backwards - Verbal Comprehension: Similarities
Digit Span: Recall of Digit Backwards - Single Word Reading (T0)
Digit Span: Recall of Digit Backwards - Single Word Spelling (T0)
Digit Span: Average - Memory: Recall of Picture

Digit Span: Average - Memory: Recall of Object (IV)
Digit Span: Average - Memory: Recall of Object (IS)
Digit Span: Average - Memory: Recall of Object (IA)
Digit Span: Average - Memory: Recall of Object (DV)
Digit Span: Average - Memory: Recall of Object (DS)
Digit Span: Average - Memory: Recall of Object (DA)
Digit Span: Average - Verbal Comprehension: Word Definitions
Digit Span: Average - Verbal Comprehension: Similarities
Digit Span: Average - Single Word Reading (T0)
Digit Span: Average - Single Word Spelling (T0)
Memory: Recall of Picture - Memory: Recall of Object (IV)
Memory: Recall of Picture - Memory: Recall of Object (IS)
Memory: Recall of Picture - Memory: Recall of Object (IA)
Memory: Recall of Picture - Memory: Recall of Object (DV)
Memory: Recall of Picture - Memory: Recall of Object (DS)
Memory: Recall of Picture - Memory: Recall of Object (DA)
Memory: Recall of Picture - Verbal Comprehension: Word Definitions
Memory: Recall of Picture - Verbal Comprehension: Similarities
Memory: Recall of Picture - Single Word Reading (T0)
Memory: Recall of Picture - Single Word Spelling (T0)
Memory: Recall of Object (IV) - Memory: Recall of Object (IS)
Memory: Recall of Object (IV) - Memory: Recall of Object (IA)
Memory: Recall of Object (IV) - Memory: Recall of Object (DV)
Memory: Recall of Object (IV) - Memory: Recall of Object (DS)
Memory: Recall of Object (IV) - Memory: Recall of Object (DA)
Memory: Recall of Object (IV) - Verbal Comprehension: Word Definitions
Memory: Recall of Object (IV) - Verbal Comprehension: Similarities
Memory: Recall of Object (IV) - Single Word Reading (T0)
Memory: Recall of Object (IV) - Single Word Spelling (T0)
Memory: Recall of Object (IS) - Memory: Recall of Object (IA)
Memory: Recall of Object (IS) - Memory: Recall of Object (DV)
Memory: Recall of Object (IS) - Memory: Recall of Object (DS)
Memory: Recall of Object (IS) - Memory: Recall of Object (DA)
Memory: Recall of Object (IS) - Verbal Comprehension: Word Definitions
Memory: Recall of Object (IS) - Verbal Comprehension: Similarities
Memory: Recall of Object (IS) - Single Word Reading (T0)
Memory: Recall of Object (IS) - Single Word Spelling (T0)
Memory: Recall of Object (IA) - Memory: Recall of Object (DV)
Memory: Recall of Object (IA) - Memory: Recall of Object (DS)
Memory: Recall of Object (IA) - Memory: Recall of Object (DA)
Memory: Recall of Object (IA) - Verbal Comprehension: Word Definitions
Memory: Recall of Object (IA) - Verbal Comprehension: Similarities
Memory: Recall of Object (IA) - Single Word Reading (T0)
Memory: Recall of Object (IA) - Single Word Spelling (T0)

Memory: Recall of Object (DV) - Memory: Recall of Object (DS)
Memory: Recall of Object (DV) - Memory: Recall of Object (DA)
Memory: Recall of Object (DV) - Verbal Comprehension: Word Definitions
Memory: Recall of Object (DV) - Verbal Comprehension: Similarities
Memory: Recall of Object (DV) - Single Word Reading (T0)
Memory: Recall of Object (DV) - Single Word Spelling (T0)
Memory: Recall of Object (DS) - Memory: Recall of Object (DA)
Memory: Recall of Object (DS) - Verbal Comprehension: Word Definitions
Memory: Recall of Object (DS) - Verbal Comprehension: Similarities
Memory: Recall of Object (DS) - Single Word Reading (T0)
Memory: Recall of Object (DS) - Single Word Spelling (T0)
Memory: Recall of Object (DA) - Verbal Comprehension: Word Definitions
Memory: Recall of Object (DA) - Verbal Comprehension: Similarities
Memory: Recall of Object (DA) - Single Word Reading (T0)
Memory: Recall of Object (DA) - Single Word Spelling (T0)
Verbal Comprehension: Word Definitions - Verbal Comprehension: Similarities
Verbal Comprehension: Word Definitions - Single Word Reading (T0)
Verbal Comprehension: Word Definitions - Single Word Spelling (T0)
Verbal Comprehension: Similarities - Single Word Reading (T0)
Verbal Comprehension: Similarities - Single Word Spelling (T0)
Single Word Reading (T0) - Single Word Spelling (T0)
Gender

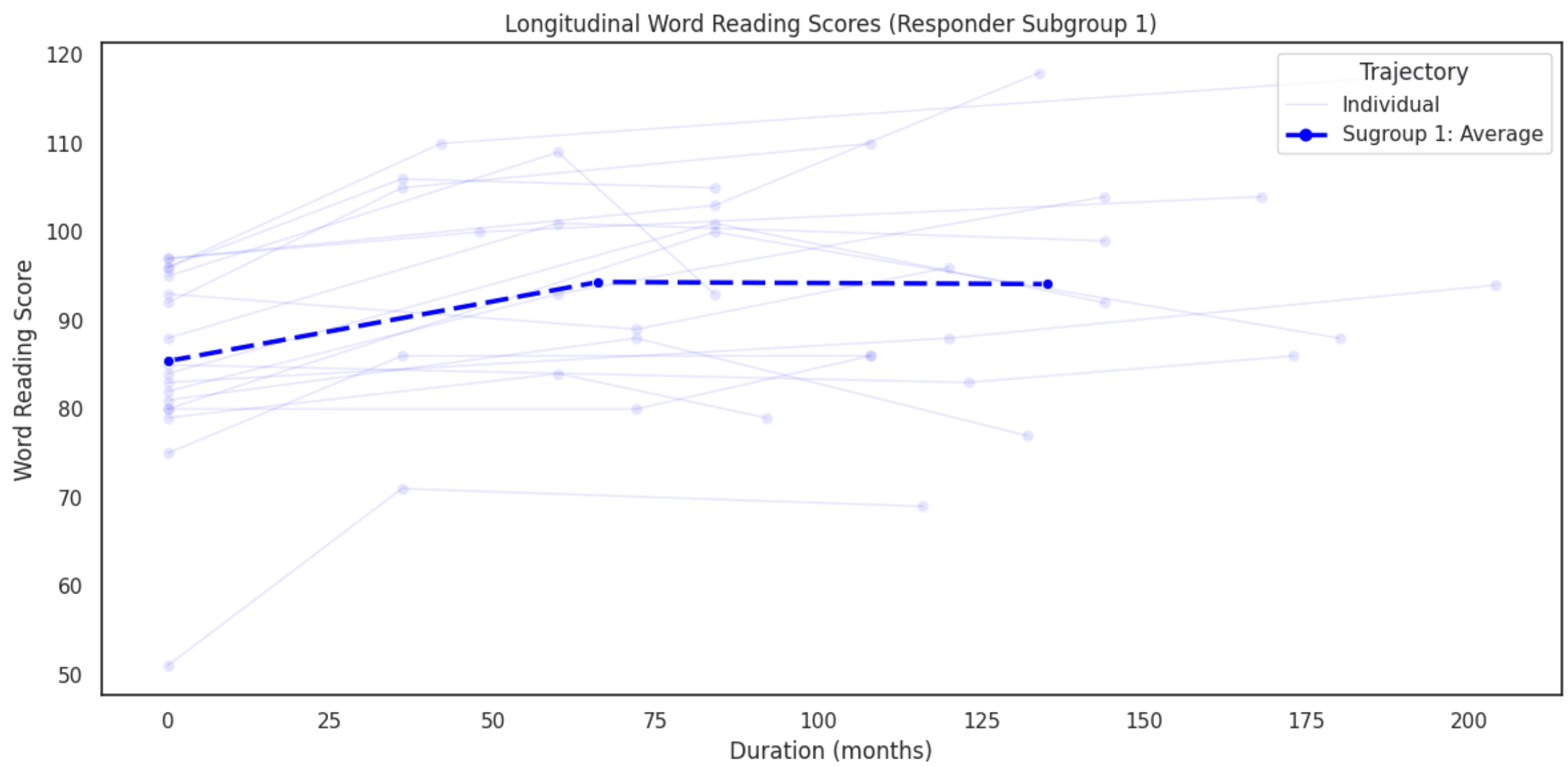

Supplementary Figure 1: Longitudinal Word Reading Scores by Responder Subgroup. The three time points are as follows: Time point 1 indicates baseline reading scores, Time point 2 indicates post intervention reading scores, Time point 3 indicates follow up reading scores.

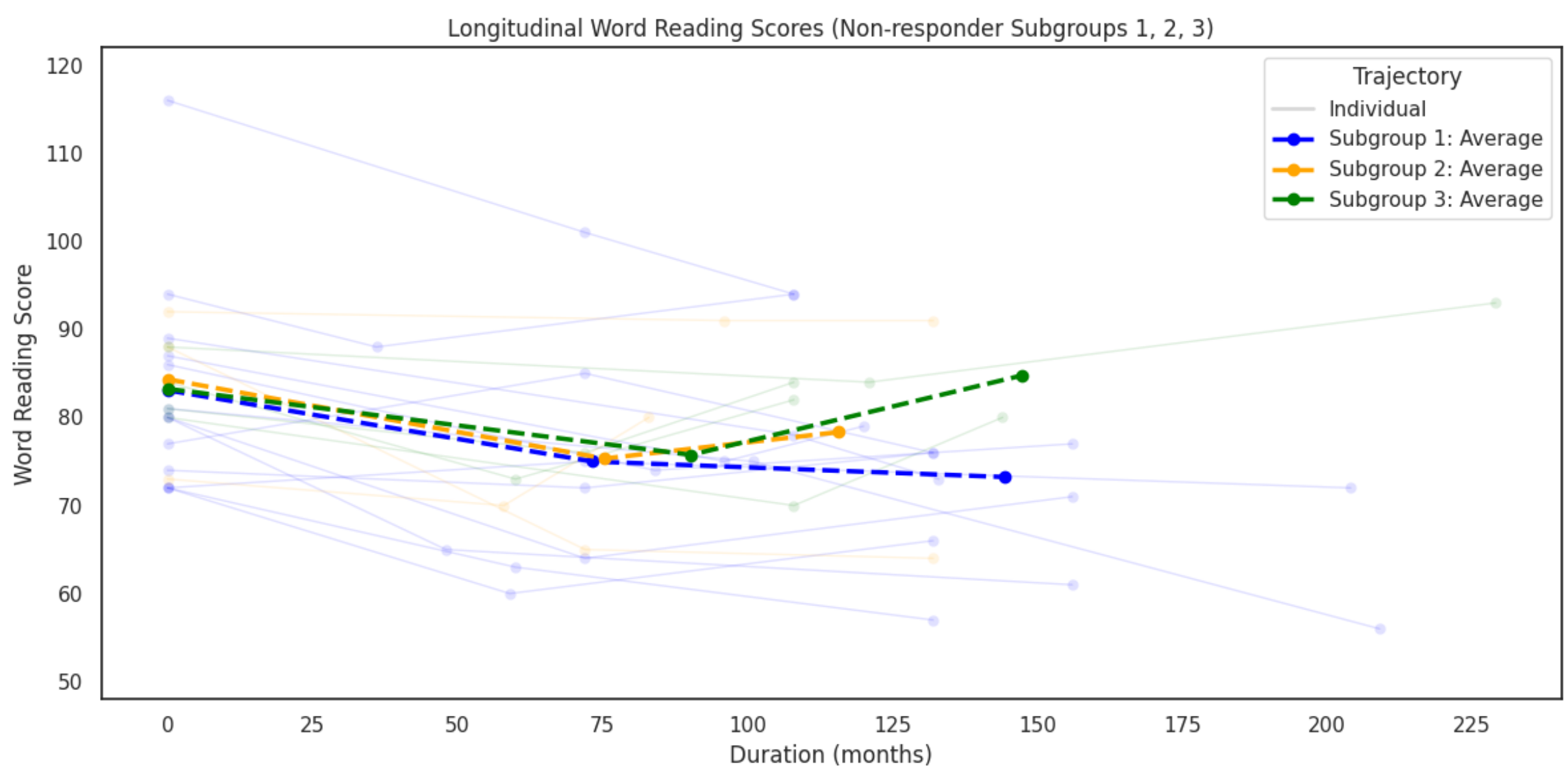

Supplementary Figure 2: Longitudinal Word Reading Scores by Non-Responder Subgroup. The three time points are as follows: Time point 1 indicates baseline reading scores, Time point 2 indicates post intervention reading scores, Time point 3 indicates follow up reading scores.

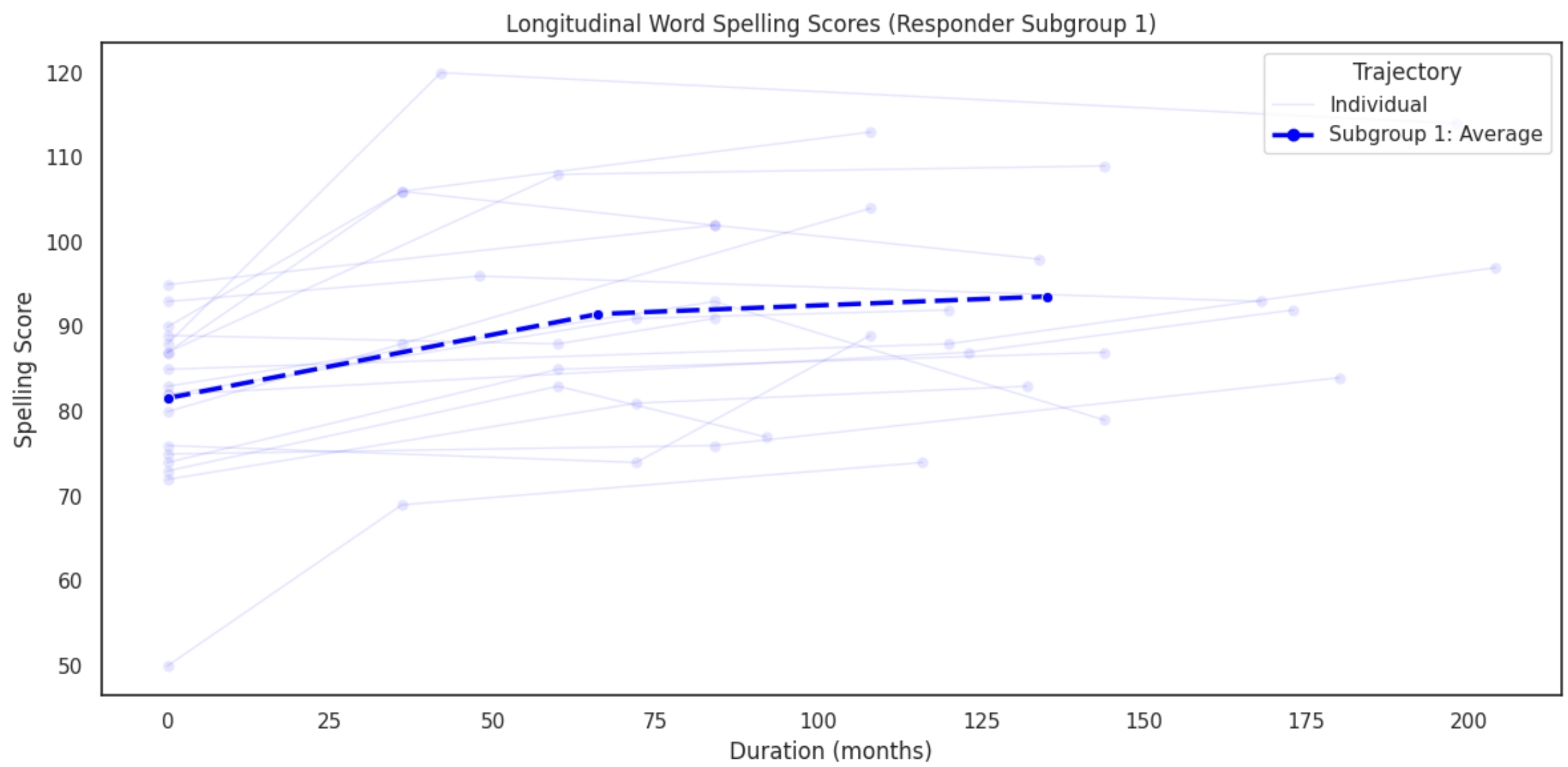

Supplementary Figure 3: Longitudinal Word Spelling Scores by Responder Subgroup. The three time points are as follows: Time point 1 indicates baseline spelling scores, Time point 2 indicates post intervention spelling scores, Time point 3 indicates follow up spelling scores.

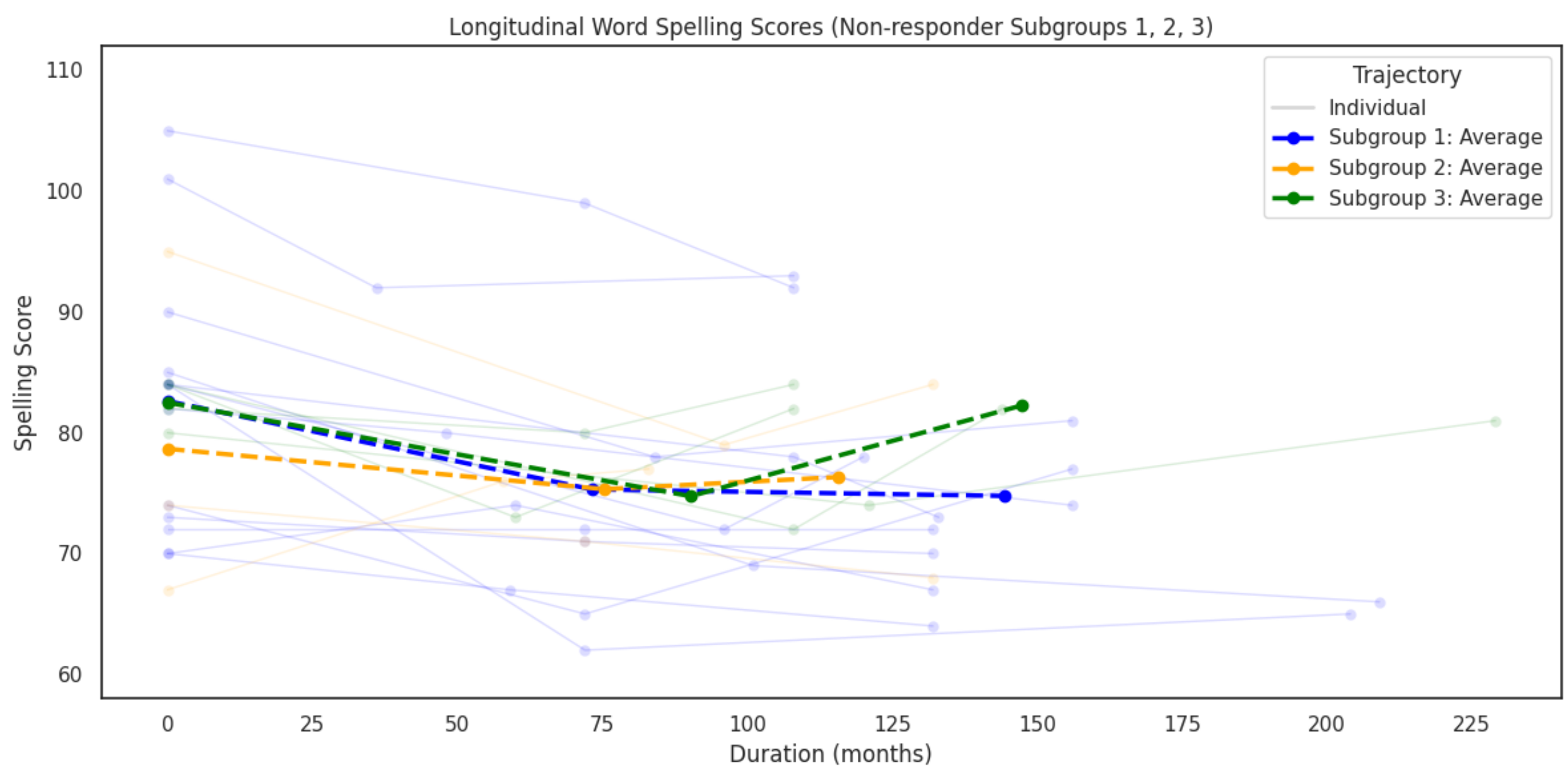

Supplementary Figure 4: Longitudinal Word Spelling Scores by Non-Responder Subgroup. The three time points are as follows: Time point 1 indicates baseline spelling scores, Time point 2 indicates post intervention spelling scores, Time point 3 indicates follow up spelling scores.

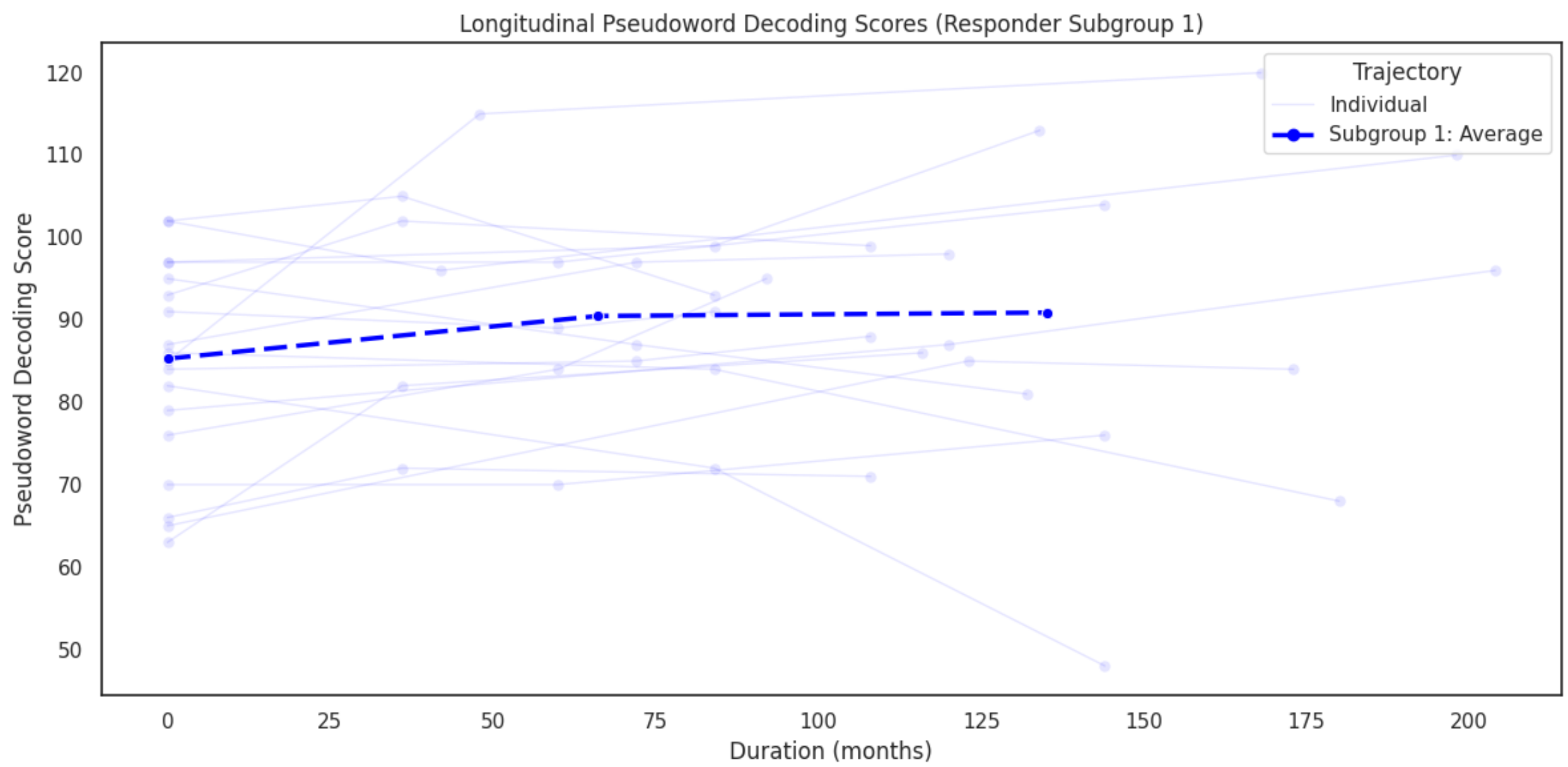

Supplementary Figure 5: Longitudinal Pseudoword Decoding Scores by Responder Subgroup. The three time points are as follows: Time point 1 indicates baseline pseudoword decoding scores, Time point 2 indicates post intervention pseudoword decoding scores, Time point 3 indicates follow up pseudoword decoding scores.

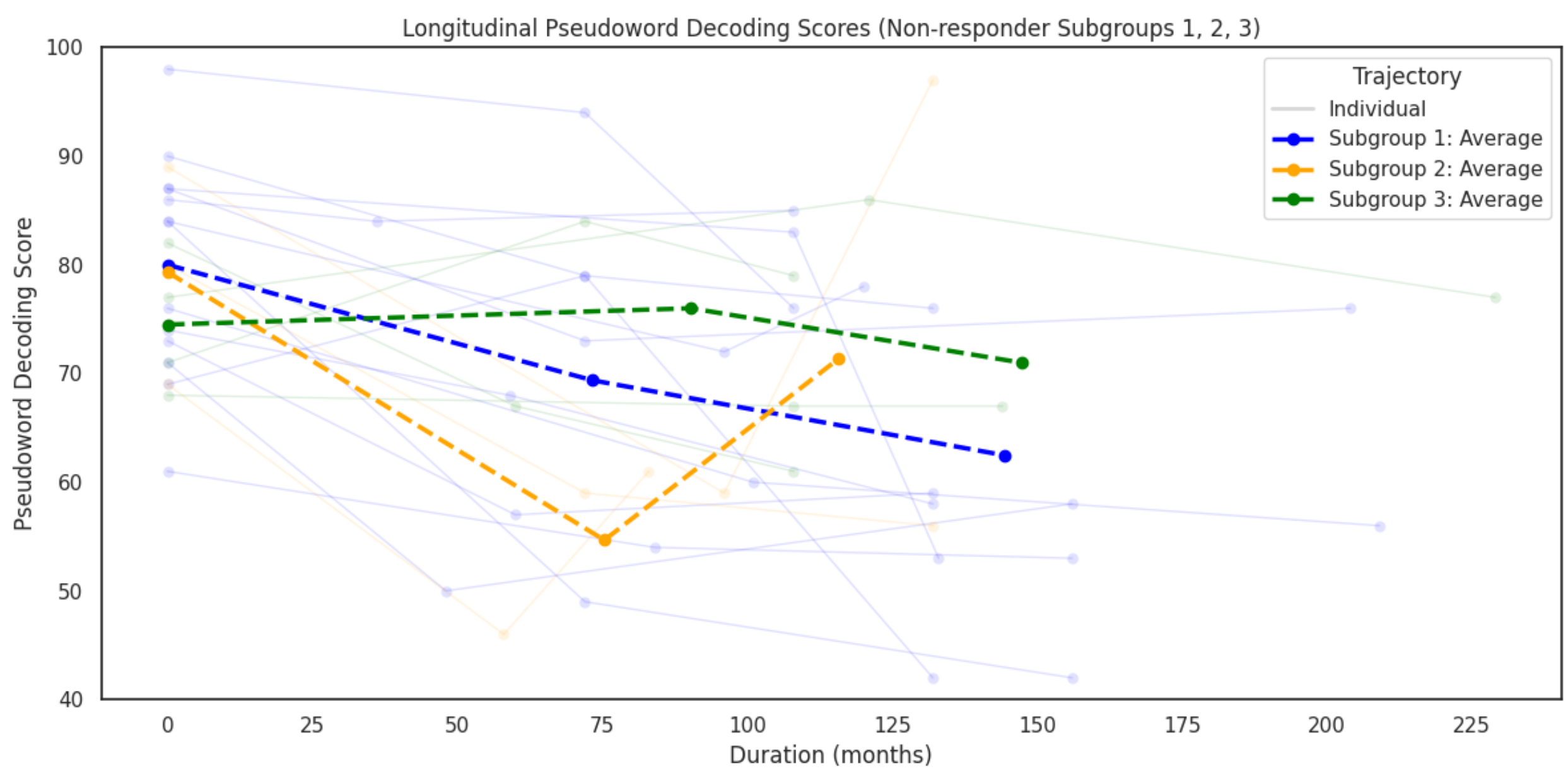

Supplementary Figure 6: Longitudinal Pseudoword Decoding Scores by Non-Responder Subgroup. The three time points are as follows: Time point 1 indicates baseline pseudoword decoding scores, Time point 2 indicates post intervention pseudoword decoding scores, Time point 3 indicates follow up pseudoword decoding scores.

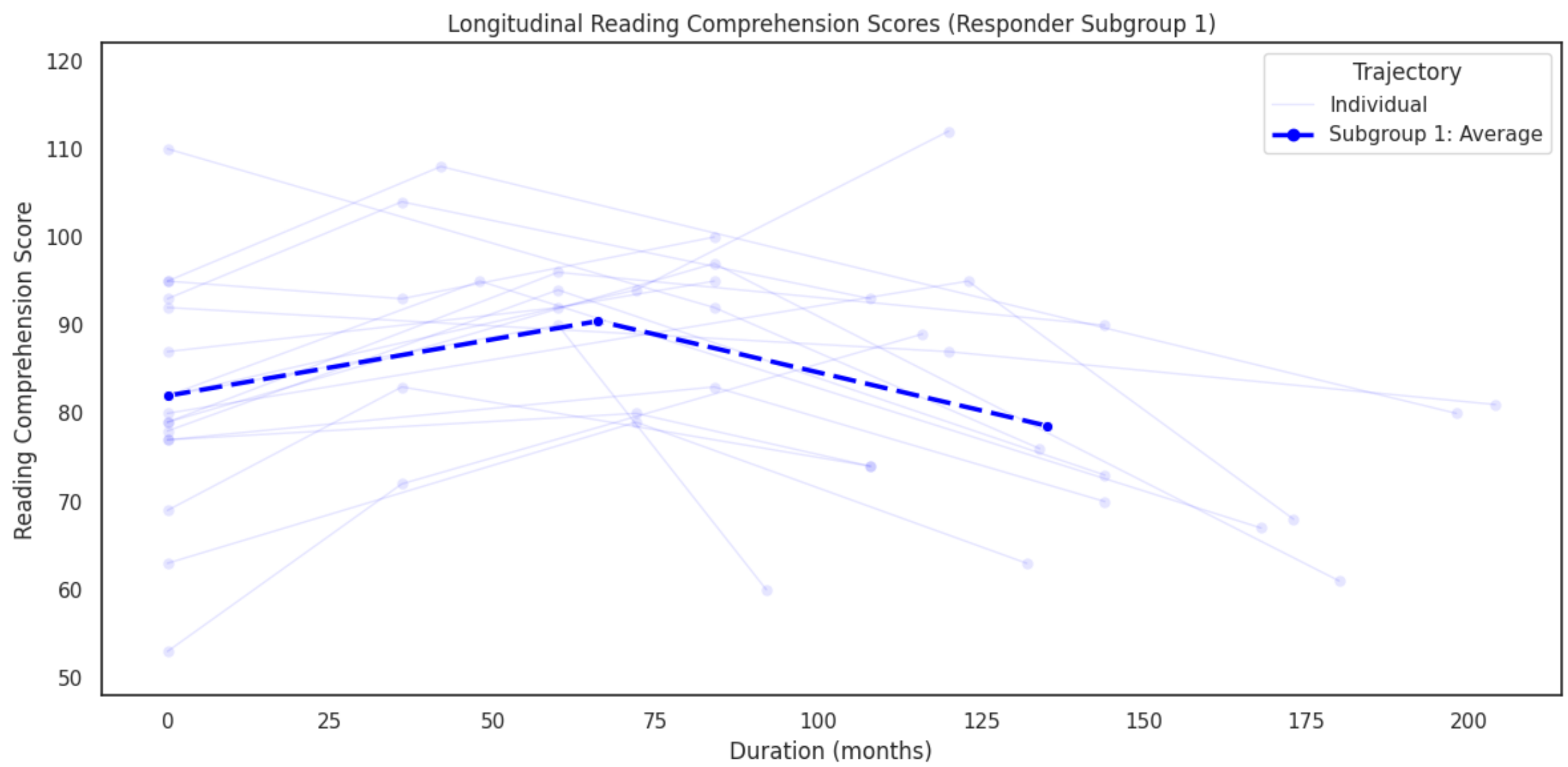

Supplementary Figure 7: Longitudinal Reading Comprehension Scores by Responder Subgroup. The three time points are as follows: Time point 1 indicates baseline reading comprehension scores, Time point 2 indicates post intervention reading comprehension scores, Time point 3 indicates follow up reading comprehension scores.

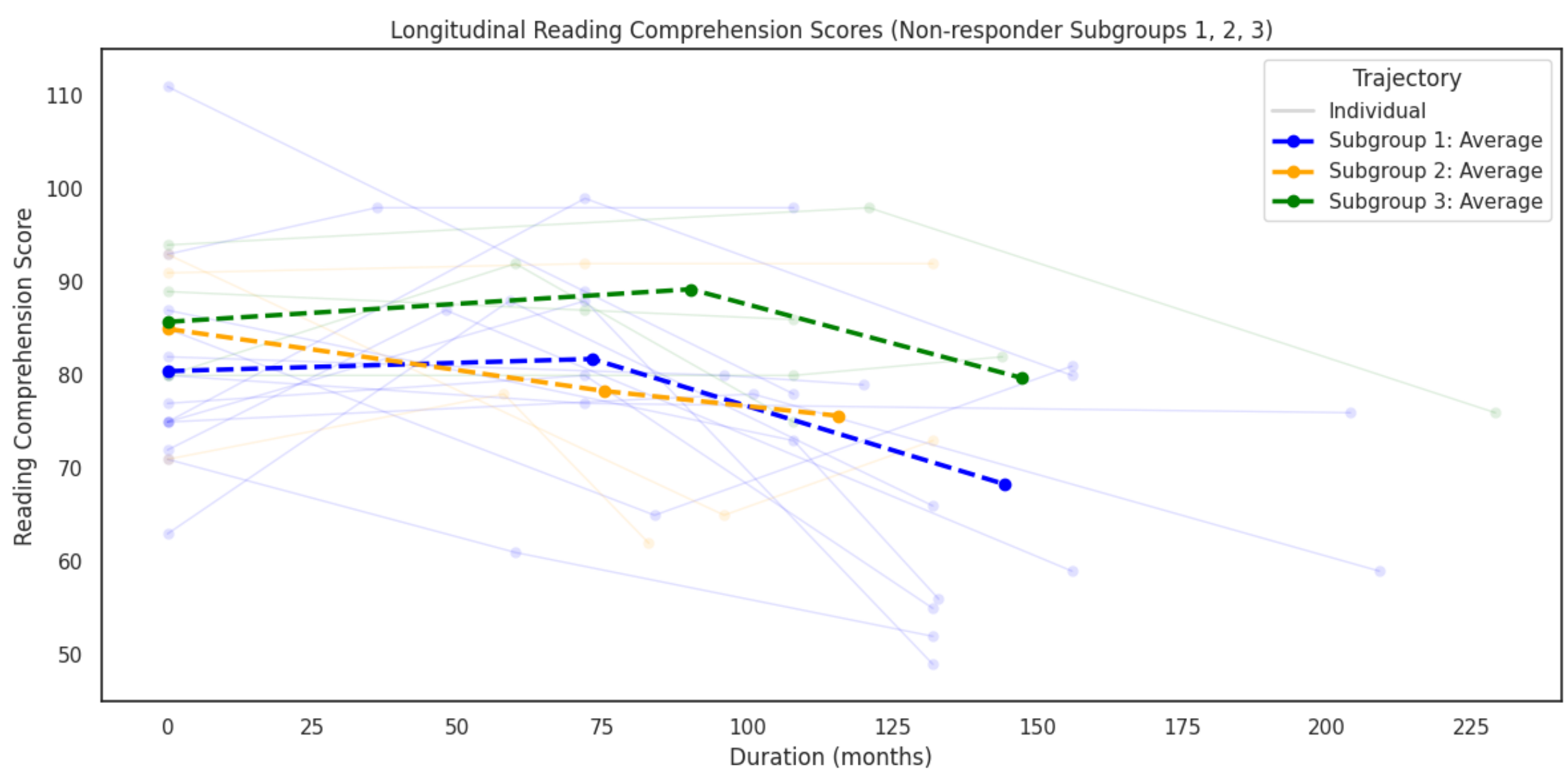

Supplementary Figure 8: Longitudinal Reading Comprehension Scores by Non-Responder Subgroup. The three time points are as follows: Time point 1 indicates baseline reading comprehension scores, Time point 2 indicates post intervention reading comprehension scores, Time point 3 indicates follow up reading comprehension scores.
